# Irisin‐Encapsulated Mitochondria‐Targeted Biomimetic Nanotherapeutics for Alleviating Acute Kidney Injury

**DOI:** 10.1002/advs.202402805

**Published:** 2024-08-09

**Authors:** Xia Zhang, Lijia Liang, Fengxian Wang, Pedro A. Jose, Ken Chen, Chunyu Zeng

**Affiliations:** ^1^ Department of Cardiology Daping Hospital Third Military Medical University (Army Medical University) Chongqing 400042 P. R. China; ^2^ Key Laboratory of Geriatric Cardiovascular and Cerebrovascular Disease Research Ministry of Education of China Chongqing 400042 P. R. China; ^3^ Chongqing Key Laboratory for Hypertension Research Cardiovascular Clinical Research Center Chongqing Institute of Cardiology Chongqing 400042 P. R. China; ^4^ Chongqing Institute of Green and Intelligent Technology Chinese Academy of Sciences Chongqing 400714 P. R. China; ^5^ Chongqing General Hospital Chongqing 401147 P. R. China; ^6^ Division of Renal Diseases & Hypertension Department of Medicine and Pharmacology‐Physiology The George Washington University School of Medicine & Health Sciences Washington DC 20037 USA

**Keywords:** acute kidney injury, biomimetic nanocarriers, irisin, mitochondria

## Abstract

Acute kidney injury (AKI) is the sudden decrease in renal function that can be attributed to dysregulated reactive oxygen species (ROS) production and impaired mitochondrial function. Irisin, a type I membrane protein secreted by skeletal muscles in response to physical activity, has been reported to alleviate kidney damage through regulation of mitochondrial biogenesis and oxidative metabolism. In this study, a macrophage membrane‐coated metal‐organic framework (MCM@MOF) is developed as a nanocarrier for encapsulating irisin to overcome the inherent characteristics of irisin, including a short circulation time, limited kidney‐targeting ability, and low membrane permeability. The engineered irisin‐mediated biomimetic nanotherapeutics have extended circulation time and enhanced targeting capability toward injured kidneys due to the preservation of macrophage membrane proteins. The irisin‐encapsulated biomimetic nanotherapeutics effectively mitigate acute ischemia‐reperfusion injury by protecting mitochondrial function and modulating SOD2 levels in renal tubular epithelial cells. The present study provides novel insights to advance the development of irisin as a potential therapeutic approach for AKI.

## Graphical abstract

A biomimetic nanotherapeutic (MCM@MOF@irisin) has been proposed to effectively mitigate acute kidney injury by enhancing the targeted delivery of irisin, a type I membrane protein secreted by skeletal muscle in response to physical activity. The objective of this study is to safeguard mitochondrial function and modulate SOD2 levels in renal tubular epithelial cells to achieve renal protection.

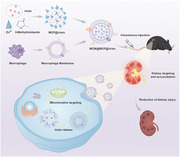

## 1 Introduction

Acute kidney injury (AKI), characterized by a sudden and rapid deterioration in renal function, contributes to the progression of chronic kidney disease (CKD) which has a high morbidity and mortality.^[^ ^1^, ^2^, ^3^ ^]^ Regrettably, effective strategies for restoring renal function following AKI are currently lacking. Renal ischemia/reperfusion (I/R) injury, accompanied by inadequate oxygen supply and excessive production of reactive oxygen species (ROS), is a leading cause of AKI.^[^ ^4^, ^5^, ^6^ ^]^ I/R‐induced mitochondrial dysfunction in renal proximal tubule cells has long been recognized as a hallmark of AKI.^[^ ^7^, ^8^ ^]^ Therefore, the preservation of mitochondrial function in I/R injury could be a promising therapeutic strategy for AKI.

Emerging evidence strongly suggests that moderate exercise provides significant benefits to patients with CKD.^[^ ^9^, ^10^ ^]^ Previous studies have revealed the involvement of irisin, a type I membrane protein secreted by skeletal muscle in response to physical activity, in mitochondrial biogenesis and oxidative metabolism.^[^ ^11^, ^12^, ^13^ ^]^ Furthermore, irisin plays a crucial role in mediating muscle‐kidney crosstalk, resulting in the mitigation of kidney damage and slowing down the progression from AKI to CKD.

Despite the proven therapeutic potential of irisin in kidney disease, the intravenous infusion of irisin is hindered by its chemical and physical instabilities within the circulation, resulting in a short half‐life.^[^ ^14^ ^]^ Moreover, intrinsic characteristics, such as decreased membrane permeability and limited organ‐specific targeting, contribute to its suboptimal therapeutic efficacy. Given the remarkable advances in nanomedicine, it is now conceivable to improve the pharmacokinetics of compounds,^[^ ^15^, ^16^ ^]^ such as irisin and its targeted delivery specifically to the kidney through intricately designed nanocarriers, thereby expediting the use of irisin for renal function restoration.

**Scheme 1:**
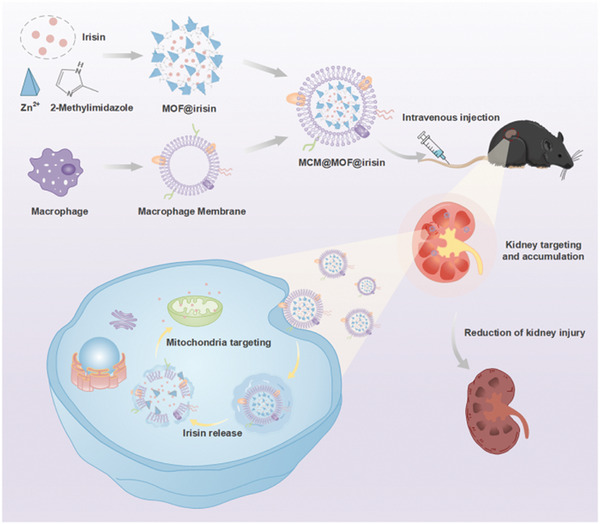
Schematic illustration of MCM@MOF@irisin biomimetic nanotherapeutics designed for targeting mitochondria in the kidney to ameliorate the acute renal injury induced by ischemia‐reperfusion.

Based on the advantages offered by cell membrane‐coated biomimetic nanocarriers, we developed a kidney‐targeted nanotherapeutics (referred to as MCM@MOF@irisin) for the treatment of AKI. We utilized a macrophage membrane‐coated metal‐organic framework (MCM@MOF) as the nanocarrier for irisin delivery, as illustrated in **Scheme** **1**. The benefit of the utilization of MOF nanocarriers for encapsulating irisin is primarily attributed to its high specific surface area, tunable pore shape and size, and biodegradability within the circulation, which holds great promise for efficient delivery of nucleic acids, siRNA, and proteins.^[^ ^17^, ^18^, ^19^, ^20^ ^]^ Importantly, the loading of irisin into the MOF nanocarrier was achieved by a one‐pot synthesis method under physiological conditions without compromising its activity.

The coating of the macrophage membrane enables evasion of phagocytosis and precise targeting of areas of inflammation, thereby prolonging the half‐life and facilitating the targeted delivery of irisin to the injured kidney. Additionally, the anti‐inflammatory properties of macrophage membrane‐coated nanoparticles may further augment the protective effects of irisin against AKI.^[^ ^21^ ^]^ In the AKI mouse model, MCM@MOF@irisin demonstrated remarkable protection against I/R‐induced renal damage by enhancing mitochondrial function. Overall, we have presented a novel irisin‐based biomimetic nanotherapeutics for mitigating AKI with great potential for clinical application.

## 2 Results

### 2.1 Preparation and Characterization of MCM@MOF@irisin

**Figure 1:**
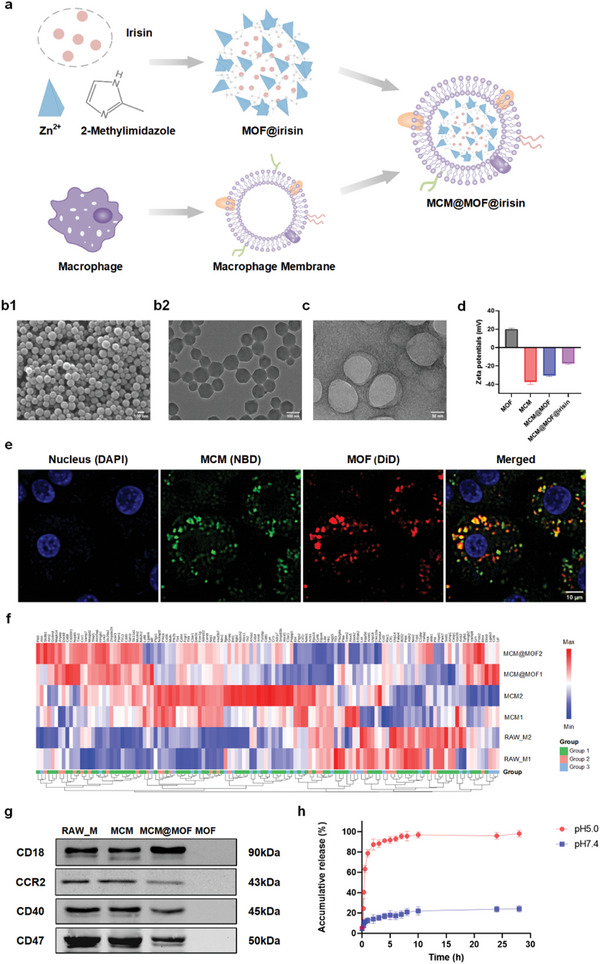
Preparation and characterization of MCM@MOF@irisin biomimetic nanotherapeutics. a) Schema of the preparation of MCM@MOF@irisin biomimetic nanotherapeutics. b) The SEM (b1) and TEM (b2) images of MOF. Scale bar = 100 nm. c) The TEM image of MCM@MOF nanocarrier. Scale bar = 50 nm. d) Zeta potentials of MOF, MCM, MCM@MOF, and MCM@MOF@irisin measured by dynamic light scattering (DLS) (n = 3 per group). e) Confocal laser scanning microscopy (CLSM) images show the colocalization of DiD‐labeled MOF nanoparticles and NBD‐labeled macrophage membrane after MCM@MOF biomimetic nanocarrier internalization in RAW264.7 cells. The colors are nucleus (blue), MCM‐NBD “shell” (green), and MOF‐DiD “core” (red). Scale bar = 10 µm. f) Heat map of proteins related to inflammation and chemotaxis detected in RAW‐M, MCM, and MCM‐MOF (n = 2 per group). Group 1 represents proteins related to inflammation. Group 2 represents proteins related to chemotaxis. Group 3 represents proteins related to both inflammation and chemotaxis. g) Western blots of CCR2 and cluster of differentiation (CD) proteins (CD18, CD40, and CD47) in RAW‐M, MCM, MCM@MOF, and MOF (n = 3 per group). (h) The release characteristics of irisin from MCM@MOF@irisin in buffers with different pH values (n = 3 per group). Data are presented as mean ± SD.

The synthesis of macrophage membrane‐coated MOF nanoparticles encapsulated with irisin, referred to as MCM@MOF@irisin, is illustrated in **Figure** **1**a. Zinc ions (Zn^2+^) and the organic ligand 2‐methylimidazole (2‐MIM) undergo self‐assembly with irisin loaded via a one‐pot approach under physiological conditions to form MOF@irisin, as previously reported.^[^ ^22^ ^]^ MOF with no irisin encapsulation was also generated using the same method. The morphology of the resulting MOF nanoparticles was examined using Scanning Electron Microscope (SEM) and Transmission Electron Microscopy (TEM), which demonstrated a monodisperse size distribution of ≈100 nm (Figure 1b). To further construct MOF‐based biomimetic nanocarrier, the generated MOF nanoparticles and purified macrophage membranes, isolated from mouse mononuclear macrophage cells (RAW264.7), were physically extruded through porous polycarbonate membranes, utilizing a previously established method.^[^ ^23^ ^]^ The optimal weight ratio of the membrane to core was determined to be 1:1 (mg:mg) throughout the entire study (Figure S1a, Supporting Information). TEM showed that the macrophage membrane layer had a superficial coverage with a mean thickness of ≈14.5 nm, thereby the MCM@MOF nanocarrier displayed an average diameter of ≈129 nm, slightly larger than that of naked MOF (Figure 1c). Moreover, the zeta potential of MCM, MCM@MOF and MCM@MOF@irisin were −37.4 mV, −30.4 mV, and −17.3 mV, respectively, as shown in Figure 1d. Subsequently, we assessed the dispersity of the nanotherapeutics and observed that the PDI values for MOF, MCM@MOF, and MCM@MOF@irisin ranged from 0.05 to 0.24 (Figure S1b, Supporting Information). This indicates that the prepared nanotherapeutics exhibit uniform size and excellent dispersion. In addition, CLSM images of RAW264.7 cells incubated with MCM@MOF for 2 h revealed the co‐localization of NBD‐labeled macrophage membrane (green) and DiD‐labeled MOF (red), as depicted in Figure 1e. This observation indicates that the core‐shell structure of the MCM@MOF nanocarrier remains intact even after cellular uptake, demonstrating the structural stability of MCM@MOF@irisin and the absence of non‐specific release of irisin. It is noteworthy that the majority of membrane proteins, including inflammation‐related and chemotactic‐related proteins, were observed to be retained within the surface of MCM@MOF, as determined by the mass spectrum analysis (Figure 1f). Notably, inflammation‐related membrane proteins, such as CD18, CCR2, CD40, and CD47 were confirmed to be well preserved on the surface of MCM@MOF nanocarrier by western blot analysis (Figure 1g), which is essential to maintain the protein‐specific targeting capability of MCM@MOF toward inflammation‐related diseases, thereby enhancing its potential for clinical applications.

The long‐term stability of the MCM@MOF nanocarrier in saline and plasma was observed over a period of two weeks. The hydrodynamic size distribution of the MCM@MOF nanocarrier remained stable in both saline and plasma, thus confirming its superior stability compared with the naked MOF (Figure S1c, Supporting Information). To determine the encapsulation efficiency and drug loading content of irisin in the MCM@MOF nanocarrier, irisin‐FITC was loaded onto MCM@MOF instead of irisin. The encapsulation efficiency and drug loading content were ultimately optimized at 94.4% and 4.7%, respectively, as determined by the fluorescence signal of FITC‐irisin (Figure S1d, Supporting Information). Nanocarriers with precise controlled release can further enhance targeted delivery and minimize toxic side effects. Previous studies have demonstrated that MOF has pH‐responsive characteristics, making it a promising nanocarrier.^[^ ^24^, ^25^, ^26^ ^]^ In order to validate the pH‐induced collapse of MOF, we subjected MOF nanoparticles to treatment with either normal physiological pH (7.4) or acidic aqueous solutions for varying durations (6 h or 24 h), followed by SEM measurements (Figure S1e, Supporting Information). We found that the degradation of MOF was accelerated under acidic conditions, while remaining stable in the normal physiological pH solution. The release of irisin from MCM@MOF@irisin was monitored under both acidic and physiological conditions for 28 h (Figure 1h). In a simulated normal physiological microenvironment with a pH of 7.4, encapsulated irisin is gradually released, amounting to less than 20% over a 28‐hour period. Due to microenvironmental acidification is generally observed in ischemic tissues,^[^ ^27^, ^28^ ^]^ the release of irisin from MCM@MOF@irisin at acidac condition was observed. The results showed an initial burst of release within the first 2 h, with ≈80% of irisin being released. This observation suggested that MCM@MOF effectively shields irisin from being released into systemic circulation, enabling targeted delivery specifically to injured organs or sites.

### 2.2 MCM@MOF@irisin Exhibited Immune‐Evasive and Kidney‐Targeting Abilities

**Figure 2:**
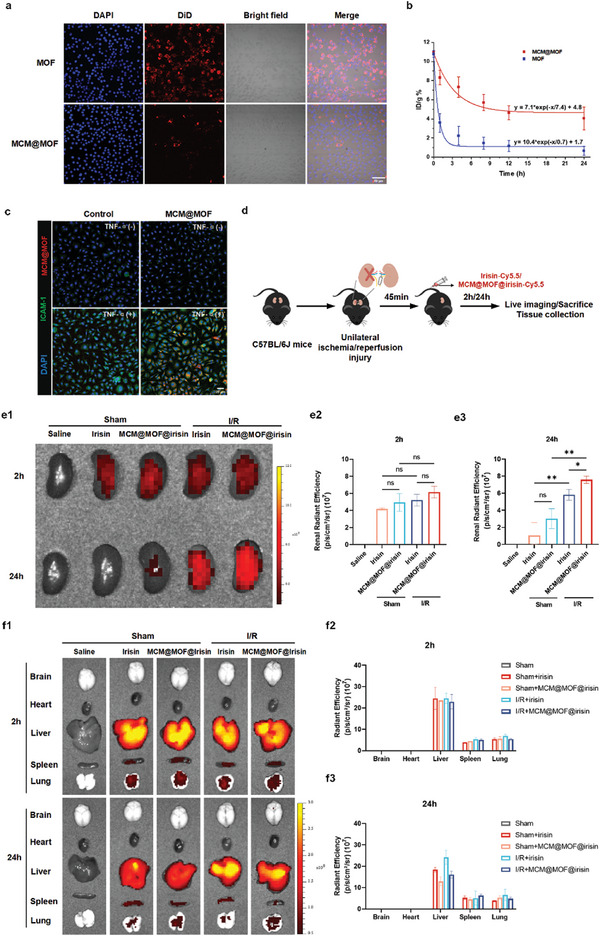
Immune‐evasive and kidney‐targeting ability of MCM@MOF@irisin nanotherapeutics. a) Confocal fluorescence images of RAW264.7 macrophages incubated with DiD‐labeled MOF or DiD‐labeled MCM@MOF for 8 h (n = 6 per group). Scale bar = 50 µm. b) Pharmacokinetics of MOF and MCM@MOF over a span of 24 h (n = 3 per group). The relative zinc content of MOF and MCM@MOF nanocarrier remaining in the blood at different time points after intravenously injection into mice via ICP‐AES measurement. c) Confocal fluorescence images of HUVECs incubated at 4 °C for 2 h in PBS (Control) or MCM@MOF nanocarrier after pretreatment with or without TNF‐α (50 ng mL ^−1^) for 6 h, MCM@MOF (red), HUVECs nucleus (blue), and ICAM‐1 (green) (n = 3 per group). Scale bar = 20 µm. d) Overview of the animal experimental design for the in vivo imaging in the unilateral renal ischemia/reperfusion injury model. e) Fluorescence images and their quantification by IVIS imaging system in kidneys from mice treated with Cy5.5 labeled‐irisin or Cy5.5 labeled‐MCM@MOF@irisin at 2 h and 24 h after their injection (n = 3 per group). Color scale, Min = 5.0 × 10^7^, Max = 1.2 × 10^8^. f) Biodistribution and quantification of irisin‐Cy5.5 and MCM@MOF@irisin‐Cy5.5 that accumulated in major organs, including the brain, heart, liver, spleen, and lung, 2 h and 24 h after their intravenous administration (n = 3 per group). Color scale, Min = 5.0 × 10^7^, Max = 3.0 × 10^8^. All images acquired with the same detection conditions, exposure time (t = 0.2 s), and excitation light power. Data are presented as mean ± SD. * P < 0.05, ** P < 0.01, ns, not significant.

To confirm the immune evasive capability of MCM@MOF@irisin, CLSM was used to evaluate the cellular uptake of the well‐designed MCM@MOF nanocarrier in the mouse macrophage cell line, RAW264.7 (**Figure** **2**a). For the CLSM analysis, both MCM@MOF and naked MOF were fluorescently labeled with DiD dye and subsequently incubated with RAW.264.7 cells. We found that MCM@MOF had reduced internalization into RAW264.7 cells compared with the naked MOF group, as evidenced by the attenuated red fluorescence signals (Figure S2a, Supporting Information). After confirming the reduction of phagocytosis by macrophages, we proceeded to investigate whether the biomimetic nanocarrier MCM@MOF can extend the half‐life of the loaded irisin. Following administration of MCM@MOF or MOF via tail vein injection, blood samples were collected from C57BL/6J mice at the specified time intervals and the cells were subsequently lysed by a mixture of nitric acid and hydrogen peroxide for Zinc content measurement by inductively coupled plasma atomic emission spectrometry (ICP‐AES). The blood circulation time of MCM@MOF was significantly longer than that of naked MOF, with half‐lives of 7.4 h and 0.7 h, respectively (Figure 2b). The previous study has demonstrated that the in vivo half‐life of irisin is less than an hour.^[^ ^14^ ^]^ This suggests that the MCM@MOF@irisin possesses enhanced blood retention properties, thereby increasing its potential for targeted delivery to injured organs and cells.

We next investigated the ability of the biomimetic nanocarrier MCM@MOF to bind to the inflamed endothelium. To establish an in vitro model,^[^ ^29^ ^]^ inflammation was induced in human umbilical vein endothelial cells (HUVECs) by tumor necrosis factor alpha (TNF‐α, 50 ng mL^−1^). Consistent with previous studies, we observed the upregulation of ICAM‐1, a key regulator of cellular inflammatory response,^[^ ^30^ ^]^ following treatment of HUVECs with TNF‐ɑ (Figure 2c). Subsequently, fluorescent‐labeled MCM@MOF nanoparticles were introduced after TNF‐ɑ stimulation to assess their inflammation targeting potential. The fluorescence signals were markedly increased in the MCM@MOF‐treated HUVECs suggesting a binding capability of MCM@MOF to inflamed endothelial cells.

Due to mass spectrum analysis revealed that the macrophage membrane is enriched with inflammatory marker proteins (Figure 1f). To investigate whether these proteins can target the damaged kidney by binding to their receptors, we conducted RNA sequencing analysis from the published database (Nephroseq), and the results are presented in Figure S3 (Supporting Information). The findings indicate that 105 proteins on the macrophage membrane have the ability to bind to 48 proteins in the damaged kidney, whose levels increased after kidney injury. Then, we explored the renal targeting efficacy of MCM@MOF@irisin using a murine model of renal ischemic/reperfusion injury. The I/R injury was induced after surgical removal of the right kidney and subsequent clamping of the left renal pedicle for a duration of 45 min, following an established protocol^[^ ^31^ ^]^ as depicted in Figure 2d. Following a 45‐minute period of ischemia, Cy5.5‐labeled MCM@MOF@irisin or irisin was administered via tail vein injection, and the fluorescence signals emitted by major organs were captured and quantified, using an IVIS. In the kidney (targeted organ), no significant differences were observed in the fluorescence signals collected from sham group treated with irisin or MCM@MOF@irisin. 2 h after I/R injury of the kidney, obvious fluorescence signals were emitted in the MCM@MOF@irisin and irisin groups, but they were not statistically different. In contrast, 24 h after I/R injury of the kidney the intensity of the fluorescence signals was significantly greater in the MCM@MOF@irisin group (1.8‐fold) than the irisin group, proving the increased accumulation of MCM@MOF@irisin in the damaged kidney (Figure 2e). Furthermore, the enhanced renal targeting ability of MCM@MOF@irisin was further validated through the examination of frozen kidney sections collected 24 h post‐injection, as shown by the increase in fluorescence signals (Figure S2b, Supporting Information). Additionally, we investigated the distribution of MCM@MOF@irisin or irisin in several organs including the brain, heart, liver, spleen, and lung (Figure 2f). It was observed that in addition to being localized in the kidney, MCM@MOF@irisin and irisin were also found in the liver. This finding may be attributed to the biological filtration function of the liver, as a majority of systemically administered nanoparticles are known to be trapped in the liver, which is consistent with previous reports.^[^ ^32^, ^33^ ^]^ The fact that the liver plays a primary role in removing circulating irisin or MCM@MOF@irisin.^[^ ^34^ ^]^ Overall, our findings demonstrated that the irisin encapsulated in the MCM@MOF nanocarrier can be efficiently delivered to the kidney, particularly following acute injury.

### 2.3 The Protective Effect of MCM@MOF@irisin Against Renal I/R Injury in Mice

**Figure 3:**
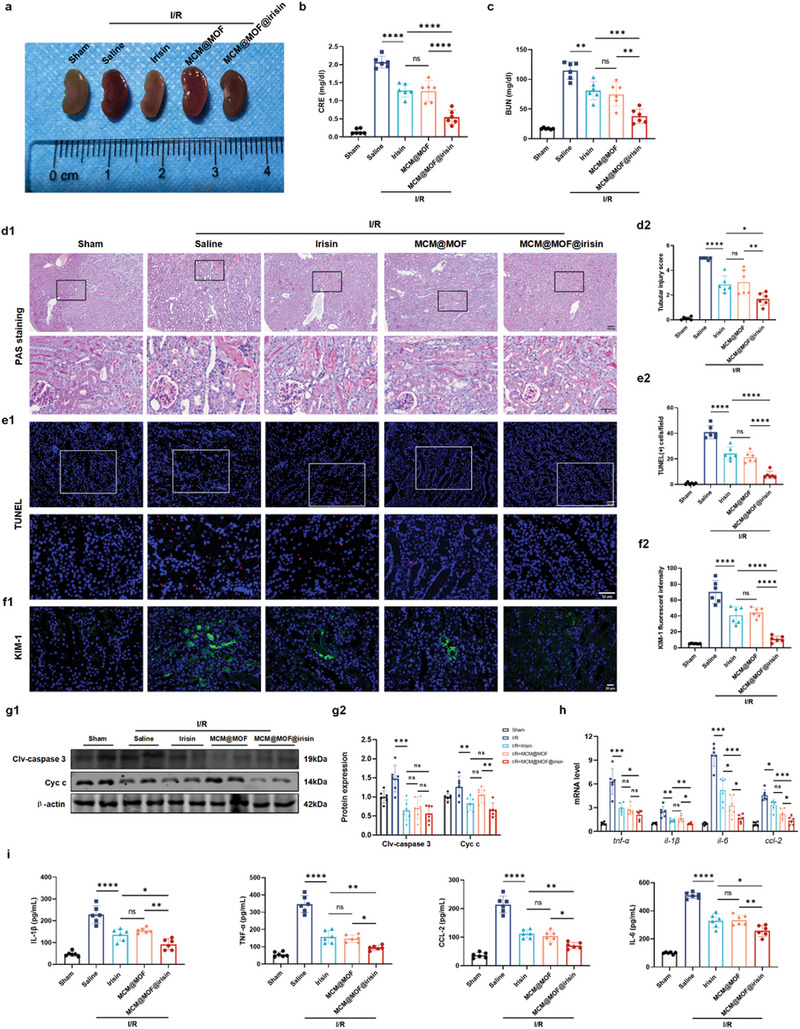
MCM@MOF@irisin nanotherapeutics against renal I/R injury in mice. a) Representative pictures of kidneys from mice with saline, irisin, MCM@MOF, and MCM@MOF@irisin treatments after renal I/R injury; sham‐operated mice treated with saline served as negative control. b,c) The levels of Scr and BUN in mice with the different treatments after 24 h (n = 6 per group). (d1) PAS staining of kidney slices of renal I/R‐injured mice with different treatments; sham‐operated mice treated with saline served as negative control (n = 6 per group). Scale bar = 100 µm. (d2) Quantification of tubular injury based on PAS staining. (e1) Renal tubular apoptosis in renal I/R‐injured mice with different treatments evaluated by TUNEL (n = 6 per group). Scale bar = 50 µm. (e2) The quantification of tubular apoptosis based on TUNEL staining. (f1) The expression of KIM‐1 in mice receiving the different treatments was assessed by immunofluorescence staining (n = 6 per group). Scale bar = 20 µm. (f2) Quantification of KIM‐1 fluorescence intensity. g) Western blot showed protein levels of cytochrome c and cleaved‐caspase 3 after different treatments in I/R injured kidneys. β‐actin was used as an internal control. h) The relative renal mRNA levels of inflammatory cytokine genes were measured by real‐time quantitative PCR (n = 6 per group). i) The levels of inflammatory factors TNF‐α, IL‐6, IL‐1β and CCL‐2 in I/R injury kidneys under different treatments. The data are presented as mean ± SD. * P < 0.05, ** P < 0.01, *** P < 0.001, **** P < 0.0001.

To assess the potential renal protective effect of MCM@MOF@irisin, AKI mice induced by I/R were intravenously injected with 0.2 mL of saline, irisin at a concentration of 5 µg mL^−1^, MCM@MOF (175 µg mL^−1^) or MCM@MOF@irisin (5 µg mL^−1^ of irisin) after a 45‐minute ischemic period. At 24 h post‐injection, blood and kidney samples were collected for further evaluation. It is evident that I/R caused substantial tissue damage, which was mitigated by irisin, MCM@MOF, and MCM@MOF@irisin treatment compared with the saline‐treated group (**Figure** **3**a).

However, MCM@MOF@irisin had a more pronounced protective effect than irisin treatment. The levels of serum creatinine (Scr) and blood urea nitrogen (BUN) in I/R mice treated with saline were significantly elevated, respectively, compared with sham mice. By contrast, the administration of MCM@MOF@irisin resulted in a much greater reduction in Scr and BUN levels as compared with irisin‐or MCM@MOF‐treated mice. These findings suggested that MCM@MOF@irisin has a remarkable efficacy in ameliorating renal function when compared with irisin (Figure 3b,c). Periodic Acid‐Schiff (PAS) staining to assess renal injury also showed that mice treated with saline exhibited severe tubular damage, characterized by the loss of brush border boundaries, detachment of tubular epithelial cells, and accumulation of cellular debris. However, treatment with irisin, MCM@MOF, or MCM@MOF@irisin resulted in a noticeable reduction in renal injury. Notably, the degree of damage observed in the MCM@MOF@irisin group was lesser than that observed in the irisin‐ and MCM@MOF‐ treated groups, indicated by tubular necrosis score (Figure 3d). We also investigated cell apoptosis using terminal deoxynucleotidyl transferase‐mediated deoxyuridine triphosphate nick‐end labeling (TUNEL) assays. We found that irisin, MCM@MOF or MCM@MOF@irisin treatment significantly attenuated the proportion of TUNEL‐positive cells in renal tissues following I/R‐induced injury. It is obvious that the group treated with MCM@MOF@irisin had superior outcomes, relative to those in the irisin‐ or MCM@MOF‐treated groups (Figure 3e). Furthermore, the protein expression of kidney injury molecule‐1 (KIM‐1), a well‐established biomarker for renal injury, had a more substantial reduction in mice treated with irisin, MCM@MOF, or MCM@MOF@irisin compared with the saline‐treated group. Remarkably, the MCM@MOF@irisin group demonstrated an even more pronounced decrease in KIM‐1 expression when compared with all the other groups (Figure 3f). Additionally, the decreased levels of cleaved‐caspase‐3 and cytochrome c were observed in the I/R mice with irisin, MCM@MOF, or MCM@MOF@irisin treatment compared with the I/R mice with saline (Figure 3g). Thus, MCM@MOF@irisin displayed superior protective effects over irisin or MCM@MOF by effectively mitigating both cellular apoptosis and KIM‐1 expression levels. Moreover, the expressions of proinflammatory cytokines, including TNF‐α, IL‐1β, IL‐6, and CCL‐2, were reduced in renal tissues collected from groups treated with either irisin or MCM@MOF@irisin (Figure 3h,i). Consistent with the other results, the decrease in the proinflammatory cytokines was much greater in the MCM@MOF@irisin than irisin or MCM@MOF treated. Overall, these findings demonstrated the superior protective effect of MCM@MOF@irisin against renal I/R injury in vivo.

### 2.4 MCM@MOF@irisin Targets Mitochondria of Renal Tubular Epithelial Cells Subjected to Hypoxia‐Reoxygenation

**Figure 4:**
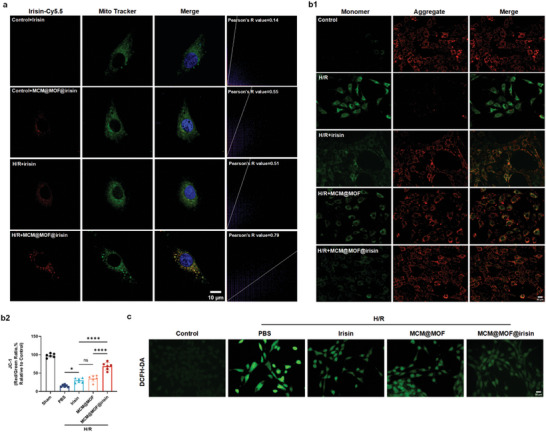
MCM@MOF@irisin protects renal tubular epithelial cells subjected to hypoxia‐reoxygenation (H/R) by ameliorating mitochondrial function. a) The mitochondrial targeting capability of irisin and MCM@MOF@irisin in HK‐2 cells under normoxia and H/R conditions observed by CLSM (n = 6 per group). The colors are nuclei (blue), MitoTracker (green), and irisin‐Cy5.5 (red). HK‐2 cells were incubated with irisin for 6 h after H/R treatment for 24 h. Scale bar = 10 µm. b) Confocal fluorescence images and quantitative analysis of mitochondrial membrane potential in HK‐2 cells stained with JC‐1 after the different treatments (n = 6 per group). Scale bar = 50 µm. c) Intracellular ROS levels were measured with DCFH‐DA fluorescence probe under different treatments. Scale bar = 50 µm. The data are presented as mean ± SD. * P < 0.05, **** P < 0.0001. ns, not significant.

Previous studies have demonstrated the crucial role of mitochondria in kidney energy metabolism and that mitochondrial dysfunction is a key factor in the pathogenesis of AKI.^[^ ^35^, ^36^, ^37^ ^]^ Additionally, exogenous irisin had been demonstrated to target mitochondria when cells were under stress condition.^[^ ^38^ ^]^ Therefore, we investigated whether MCM@MOF@irisin has enhanced mitochondrial targeting capability that could further benefit its therapeutic effect. HK‐2 cells subjected to hypoxia/reoxygenation (H/R) stress were incubated with Cy5.5‐ labeled irisin or MCM@MOF@irisin, while HK‐2 cells incubated with Cy5.5‐ labeled irisin or MCM@MOF@irisin under normoxia served as negative controls. It can be seen that irisin can target and accumulate in the mitochondria of cells under H/R but not under normoxia conditions. MCM@MOF@irisin exhibited enhanced mitochondrial targeting ability compared with irisin under normal or stress condition, as evidenced by a significantly higher degree of the localization within the mitochondria for MCM@MOF@irisin than irisin alone (**Figure** **4**a). The change in mitochondrial membrane potential was subsequently assessed using the JC‐1 fluorescence probe to validate the protective effect of irisin, MCM@MOF, and MCM@MOF@irisin in preserving mitochondrial function. It was observed that following H/R stimulation, HK‐2 cells exhibited a significant increase in red fluorescence (JC‐1 aggregate), indicating H/R‐induced damage to their mitochondria. However, treatment with irisin or MCM@MOF for 6 h notably enhanced the ratio of JC‐1 red/green in HK‐2 cells, which was more pronounced with MCM@MOF@irisin treatment (Figure 4b). Furthermore, the levels of ROS in HK‐2 cells subjected to H/R were assessed using DCFH‐DA fluorescence probe (Figure 4c). It is evident that lowest levels were observed in the MCM@MOF@irisin group compared to all other groups. Consistently, MCM@MOF@irisin exhibited a more pronounced improvement in cell survival of H/R injured HK‐2 cells compared with irisin alone (Figure S4a,b, Supporting Information). Additionally, the activity of catalase (CAT) and levels of malondialdehyde (MDA) were further investigated (Figure S4c,d, Supporting Information). It was observed that MCM@MOF@irisin enhanced the activities of CAT and decreased the MDA levels more significantly than irisin or MCM@MOF treated. These findings show that intervention with MCM@MOF@irisin effectively stabilizes mitochondrial function and mitigates oxidative stress damage in vitro.

### 2.5 MCM@MOF@irisin Nanotherapeutics Maintain Mitochondrial Function In Vivo

**Figure 5:**
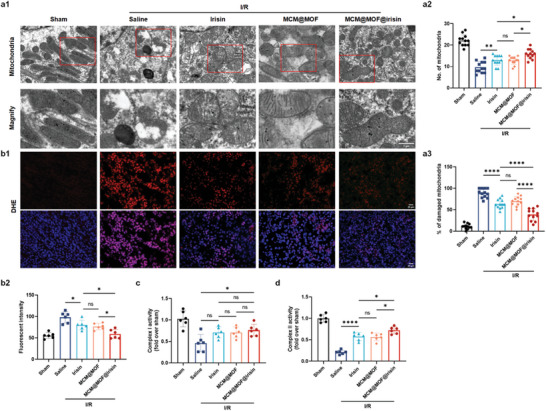
MCM@MOF@irisin protected mitochondria from damage in renal I/R injured mice. a) Bio‐TEM images of mitochondrial injury and percentage of damaged mitochondria in mice kidneys (n = 3 per group). Scale bar = 1 µm. b) Fluorescence images and quantification of oxidative stress, using DHE staining, in kidney slices of I/R injured mice with different treatments (n = 6 per group). Scale bar = 20 µm. c,d) Mitochondrial respiratory chain complex I and II enzymatic activity in I/R injury kidneys (n = 6). The data are presented as mean ± SD. * P < 0.05, ** P < 0.01, **** P < 0.0001.

Bio‐TEM imaging was performed to evaluate renal injury by examining the structure of mitochondria. The results revealed that compared with sham mice, the mitochondria in I/R‐injured mice exhibited swelling with decreased cristae and even rupture with disrupted membranes, indicating extensive and severe damage caused by I/R injury. However, treatment with irisin, MCM@MOF, and MCM@MOF@irisin effectively attenuated mitochondrial damage, resulting in a reduction in the number of damaged mitochondria in mice. Moreover, the protective effect on mitochondria was stronger in MCM@MOF@irisin than irisin treatment (**Figure** **5**a1–a3). Furthermore, the ROS levels in renal tissues were assessed using DHE staining in mice subjected to different treatments, as shown in Figure 5b. Remarkably, treatment with irisin resulted in a significant reduction in ROS levels within renal tissue, with slightly lower levels observed in the MCM@MOF@irisin‐treated group compared with the irisin‐ or MCM@MOF‐treated groups. Furthermore, in order to confirm whether irisin can maintain mitochondrial function by restoring the activity of the electron transport chain, we assessed renal mitochondrial function in vivo by evaluating the activities of complex I and complex II (Figure 5c,d). The results showed a significant reversal of the activities of complex I and complex II in I/R mice following treatment with MCM@MOF@irisin. Additionally, the reduced CAT activities and elevated MDA levels in I/R mice were reversed by treatment with MCM@MOF@irisin or irisin, although the effect was more pronounced with MCM@MOF@irisin than with irisin (Figure S5a,b, Supporting Information). Collectively, these findings demonstrate that MCM@MOF@irisin protects against tubular injury by improving mitochondrial function and reducing oxidative stress‐induced damage.

### 2.6 The Protective Effect of MCM@MOF@irisin Nanotherapeutics on I/R Injury Reduces Progression of AKI to CKD

**Figure 6:**
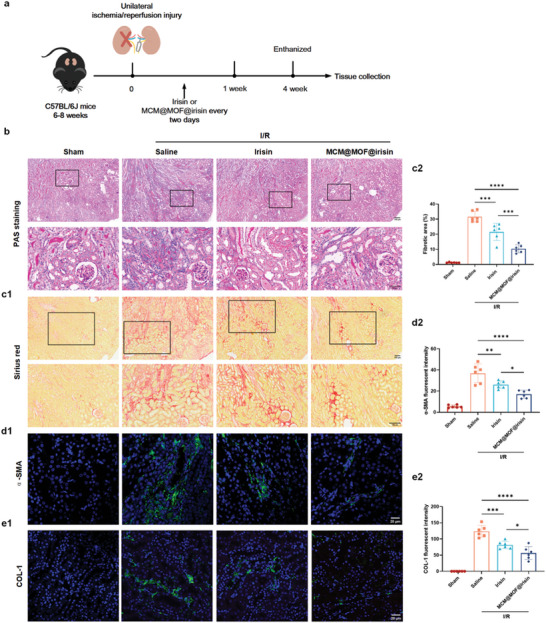
MCM@MOF@irisin mitigated fibrogenesis and the progression of CKD in kidneys of mice after unilateral ischemia/reperfusion injury. a) Treatment protocol of the renal I/R injury mice model. b) PAS staining of kidney slices of renal I/R‐injured mice with different treatments; sham‐operated mice treated with saline served as negative control (n = 6 per group). Scale bar = 100 µm. (c1) Representative images of Sirius red staining, in kidney slices of renal I/R‐injured mice with different treatments (n = 6 per group). Scale bar = 100 µm. (c2) Quantification of fibrotic area based on Sirius red staining. d,e) Expression of the fibrotic marker α‐SMA, COL‐1 in kidney slices of renal I/R‐injured mice (n = 6 per group). Scale bar = 20 µm. The data are presented as mean ± SD. * P < 0.05, ** P < 0.01, *** P < 0.001, **** P < 0.0001.

To further validate the protective effect of MCM@MOF@irisin nanotherapeutics on I/R injury to reduce the progression of AKI to CKD, saline, irisin, or MCM@MOF@irisin was intravenously administered in I/R mice four times during the first week with a two‐day interval between each administration. After 4 weeks of treatment, the kidneys were collected for evaluation (**Figure** **6**a). PAS and Sirius red staining revealed significant attenuation of kidney injury and interstitial fibrosis in I/R‐induced AKI mouse models treated with either irisin or MCM@MOF@irisin compared with the saline group (Figure 6b,c). Notably, MCM@MOF@irisin exhibited superior therapeutic efficacy relative to irisin. Immunohistochemical analysis consistently demonstrated marked reduction in α‐SMA (a fibrosis marker) and type I collagen (COL‐1) expression levels within the kidney at day 28 post‐AKI following treatment with MCM@MOF@irisin compared with saline‐ or irisin‐treated renal I/R‐injured mice (Figure 6d,e). Collectively, these findings suggest that MCM@MOF@irisin holds promise as a potential treatment for ameliorating I/R Injury.

### 2.7 Biocompatibility of MCM@MOF In Vivo

**Figure 7:**
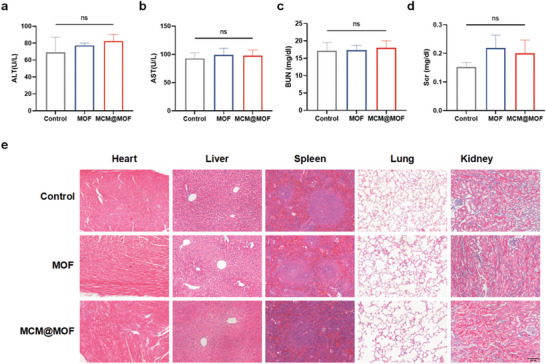
The biosafety evaluation of MCM@MOF nanocarrier. a–d) ALT, AST, BUN, and Scr levels in blood of mice after the intravenous injection of MOF or MCM@MOF (n = 3 per group). e) H&E‐stained histological sections from major organs, 21 days after the intravenous administration of saline, MOF, or MCM@MOF into healthy mice (n = 3 per group). Scale bar = 100 µm. The data presented as mean ± SD. ns, not significant.

The potential in vivo toxicity of nanomaterials is always of great concern, necessitating further assessment of the adverse effects of nanocarriers before their clinical translation. C57BL/6J mice were intravenously injected with a single dose of 1 mg kg ^−1^ MCM@MOF and MOF every other day for 21 days, while saline‐injected mice served as a control group. Blood samples and major organs were collected for blood biochemical and histological analysis. It can be seen that MCM@MOF and MOF had no effects on hepatic or renal function (**Figure** **7**a–d), as indicated by the levels of serum alanine aminotransferase (ALT), aspartate aminotransferase (AST), Scr, and BUN. Furthermore, no significant histological damage to major organs such as the heart, liver, spleen, lung, and kidney was observed in H&E images (Figure 7e). We further assessed the acute in vivo toxicity of the MCM@MOF nanocarrier. C57BL/6J mice were intravenously injected a single dose of 10 mg kg ^−1^ in MCM@MOF or MOF. After 24 h of injection, blood samples and major organs were collected for analysis. Remarkably, there were no discernible alterations in MCM@MOF‐ and MOF‐treated mice compared to control mice (Figure S6a‐c, Supporting Information). These findings suggested the excellent biocompatibility of the MCM@MOF nanocarrier.

### 2.8 The Protection of MCM@MOF@irisin on Mitochondria via Modulation of SOD2

**Figure 8:**
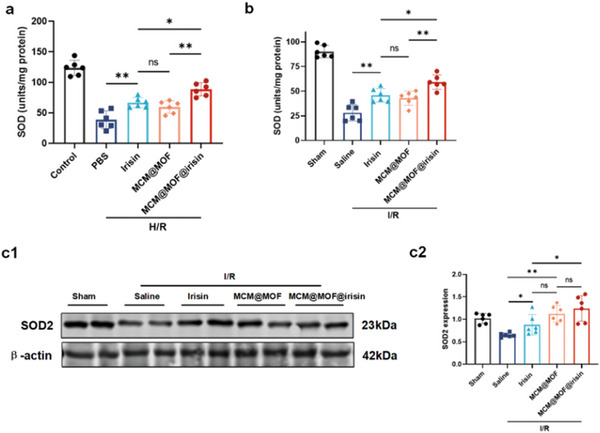
The protection of MCM@MOF@irisin on mitochondria via modulation of SOD2. a) SOD activities of H/R injured HK‐2 cells after incubation in PBS, irisin, MCM@MOF, or MCM@MOF@irisin; HK‐2 cells incubated with PBS under normoxia served as control (n = 6 per group). b) SOD activities of kidneys after treatment with saline, irisin, MCM@MOF, or MCM@MOF@irisin (n = 6 per group). c) Western blot analysis of SOD2 expression in the kidneys of AKI mice following treatment with saline, irisin, MCM@MOF, or MCM@MOF@irisin (n = 6 per group). β‐actin served as a loading control.

SOD has been found to have a protective effect on the kidney and is able to maintain normal kidney function.^[^ ^39^ ^]^ Studies have demonstrated that SOD can effectively reduce kidney damage and improve kidney function in patients with kidney disease.^[^ ^40^ ^]^ In addition, our previous study has demonstrated that irisin can protect mitochondrial function against myocardial infarction by regulating superoxide dismutase (SOD).^[^ ^41^ ^]^ Therefore, we proposed that MCM@MOF@irisin may restore mitochondrial function by preserving SOD activity. We evaluated the SOD activity in HK‐2 cells exposed to H/R, and observed a H/R‐induced decrease in SOD enzymatic activity (**Figure** **8**a), which was reversed by irisin, MCM@MOF, or MCM@MOF@irisin treatment. The similar results were got in the in‐vivo experiment, I/R‐induced SOD activity was reversed by irisin, MCM@MOF, or MCM@MOF@irisin treatment (Figure 8b). These results suggest that MCM@MOF@irisin may have the potential to protect mitochondrial function against renal I/R injury by enhancing SOD activities.

The kidneys contain three subtypes of SOD: SOD1, SOD2, and SOD3. Among these, SOD2 is predominantly located in the mitochondria.^[^ ^44^ ^]^ It has been observed that irisin or MCM@MOF@irisin can target the mitochondria and protect mitochondrial function. Therefore, we checked the role of SOD2 in the irisin‐mediated protection in kidney against I/R injury. Western blot analysis the decreased SOD2 expression by I/R was reversed by irisin, MCM@MOF, or MCM@MOF@irisin treatment (Figure 8c). The regulation of irisin on SOD2 was occurred in the post‐transcriptional levels, because irisin or MCM@MOF@irisin increased SOD2 protein, not mRNA expression (Figure S7, Supporting Information). Notably, MCM@MOF@irisin demonstrated a superior effect on SOD2 expression than irisin alone.

## 3 Discussion

In the present study, we have demonstrated a novel irisin‐mediated biomimetic nanotherapeutics that effectively mitigates I/R‐induced AKI. The developed biomimetic nanocarrier, constructed using a macrophage membrane‐coated metal‐organic framework, successfully encapsulates irisin and overcomes its inherent limitations, such as short circulation time, limited kidney‐targeting ability, and low membrane permeability. In a renal I/R mouse model, the MCM@MOF@irisin nanotherapeutics significantly ameliorates AKI symptoms, in part by enhancing mitochondrial function. These findings suggest that our developed biomimetic nanotherapeutics holds great promise as a potential therapeutic intervention for AKI.

Accumulating evidence suggests that moderate exercise confers significant benefits to patients with CKD.^[^ ^9^ ^]^ Previous studies have demonstrated that irisin, secreted by skeletal muscles in response to physical exercise, plays a pivotal role in mediating the crosstalk between muscle and kidney, thereby attenuating renal damage in AKI and CKD.^[^ ^45^, ^46^ ^]^ Despite the therapeutic potential of irisin in kidney damage, its clinical applications are hindered by inherent limitations such as a short half‐life, low membrane permeability, and limited organ‐specific targeting.

Nanomaterials can serve as carriers for encapsulating drugs, thereby improving the pharmacokinetics of peptide‐ or protein‐based medications. This enhancement reduces their susceptibility to physiological influences and enhances their therapeutic effect.^[^ ^47^, ^48^ ^]^ Additionally, in some cases, nanomaterials themselves may possess therapeutic properties.^[^ ^49^, ^50^ ^]^ However, owing to their exogenous nature, these nanocarriers are more susceptible to recognition and phagocytosis by the immune system, resulting in an accelerated drug clearance rate and a diminished therapeutic effect. Biomimetic nanomaterials, particularly cell‐membrane coated nanoparticles, possess the combined advantages of nanoparticles and functional cells. These biomimetic nanocarriers exhibit enhanced capabilities in terms of blood circulation, immune evasion, and targeted drug delivery, thereby garnering significant interest in the field.^[^ ^23^, ^51^, ^52^ ^]^ Among the cellular components of the innate immune system, macrophages and neutrophils are the primary cells that respond to pathogens in the body. Neutrophils have a lifespan of less than 24 h in most cases, while macrophages can survive for several months or even longer.^[^ ^53^, ^54^ ^]^ Additionally, macrophages possess active targeting capabilities, high immune compatibility, and prolonged circulation time. Considering that renal I/R injury can induce a severe inflammatory response, we hypothesized that employing a macrophage membrane‐coated nanocarrier could potentially encapsulate irisin, thereby prolonging its circulation time and enhancing targeted delivery to the injured kidney. In this study, we developed the biomimetic nanotherapeutics, MCM@MOF@irisin, by macrophage membrane‐coated metal‐organic frameworks with loaded irisin. Our in‐vitro investigations confirmed that this nanotherapeutics possess immune evasion capabilities. Moreover, MCM@MOF@irisin effectively extended the half‐life of irisin from less than one hour to 7.4 h. Additionally, our results demonstrated that MCM@MOF exhibited binding affinity toward inflamed endothelial cells in vitro and displayed significant accumulation at the site of renal injury in vivo. Collectively, these findings highlight the therapeutic potential of our developed MCM@MOF@irisin nanotherapeutics to the advancement of irisin therapy.

To evaluate the in‐vivo protective efficacy of MCM@MOF@irisin, we utilized a mouse model of renal I/R injury. Utilizing this model, we observed that MCM@MOF@irisin nanotherapeutics exhibited superior effectiveness in protecting renal function compared with irisin or MCM@MOF, as evidenced by the levels of Scr and BUN. Histological analysis revealed that I/R‐induced renal damage was characterized by severe tubular injury, which was significantly reduced by MCM@MOF@irisin treatment. Furthermore, MCM@MOF@irisin significantly mitigated renal tissue apoptosis following injury induced by I/R. Moreover, the reduction in KIM‐1, a well‐established biomarker for renal injury, and proinflammatory cytokines in mice treated with MCM@MOF@irisin was more pronounced compared with those treated with irisin or saline. Accumulating evidence suggested that maladaptive changes in the kidneys following AKI contribute to the development of CKD. In the present study, we found the protection of irisin in the acute phase yielded a long‐term outcome by reduction of AKI progress to CKD.

Mitochondrial dysfunction plays a pivotal role in the pathogenesis of AKI.^[^ ^35^ ^]^ In our previous study, we demonstrated that circulating irisin can ameliorate lung I/R injury by preserving mitochondrial function.^[^ ^38^ ^]^ However, due to its negative charge, irisin has difficulty in penetrating both plasma and mitochondrial membranes. Even with increased membrane permeability during I/R conditions, the penetration of irisin remains limited. Herein, we demonstrate that MCM@MOF@irisin effectively enhances the mitochondrial‐targeting ability of irisin irrespective of normal or stress conditions, thereby augmenting its therapeutic efficacy. In vitro studies confirmed that MCM@MOF@irisin preserves mitochondrial function, including maintenance of mitochondrial membrane potentials, CAT activities, and MDA levels. Additionally, bio‐TEM imaging revealed a significant reduction in the number of damaged mitochondria upon treatment with MCM@MOF@irisin. Furthermore, effective mitigation of oxidative stress, a major contributor to mitochondrial damage, was observed. The activities of complex I and complex II reverse by MCM@MOF@irisin also were observed. Overall, our data substantiate the effectiveness of MCM@MOF@irisin in reducing I/R‐induced renal injury by enhancing the mitochondrial function.

Our previous study has demonstrated that irisin can maintain mitochondrial function by modulating SOD2 levels against injured myocardium.^[^ ^41^ ^]^ Given the co‐localization of irisin or MCM@MOF@irisin with mitochondria in this study, along with the known localization of SOD2 in the mitochondria,^[^ ^44^ ^]^ we investigated the role of SOD2 in irisin‐mediated protection in the kidney against I/R injury. Western blot analysis of SOD2 expression showed that MCM@MOF@irisin had a superior effect on SOD2 expression compared to irisin alone. Furthermore, we conducted an assessment of the SOD activity in HK‐2 cells subjected to H/R as well as in mice undergoing I/R. The findings revealed a reduction in SOD enzymatic activity; however, this effect was found to be reversible following treatment with irisin, MCM@MOF, or MCM@MOF@irisin. Given the accumulating evidence demonstrating SOD2 as a specific target of SIRT3, and the pivotal role played by SIRT3 in promoting SOD2 activity through the regulation of SOD2 acetylation sites to maintain mitochondrial functions,^[^ ^42^, ^43^ ^]^ it is worth noting that the mRNA expression of SIRT3 in AKI mice can be upregulated following treatment with irisin, MCM@MOF or MCM@MOF@irisin (data not shown). This suggests that SIRT3 may mediate the regulation of SOD2 activity under stress. Further investigations into how exactly SIRT3 promotes SOD2 activity through de‐acetylation are warranted for future research.

Additionally, previous studies have demonstrated that irisin exhibits inhibitory effects on pyroptosis,^[^ ^55^ ^]^ necrosis,^[^ ^56^ ^]^ and ferroptosis.^[^ ^57^ ^]^ This suggests that irisin may offer protection through multiple pathways. Future studies can further investigate the mechanisms by which MCM@MOF@irisin protects against renal I/R injury. Moreover, our study found that MCM@MOF@irisin can also accumulate in the liver and lung, in addition to its target organ, the kidney. Previous studies have shown that irisin has a protective effect on liver and lung injuries.^[^ ^38^, ^56^ ^]^ Therefore, we expect that our developed MCM@MOF@irisin can be more widely utilized in diseases related to the liver and lungs. Further research will be pursued in the future.

## 4 Conclusion

In conclusion, we have proposed a kidney‐targeted MCM@MOF@irisin biomimetic nanotherapeutic for the treatment of AKI induced by I/R. Our comprehensive investigations demonstrated that the biomimetic nanocarrier confers the encapsulated irisin with increased circulation time, and enhanced kidney‐targeting efficacy, and subcellular delivery, and exhibiting significant protection against I/R‐induced renal injury. Further analysis revealed that irisin‐mediated biomimetic nanotherapeutics can effectively ameliorate mitochondrial function under I/R stress conditions. Overall, our current work has established a promising nanotechnology strategy to alleviate effectively AKI and oxidative stress, demonstrating remarkable potential for clinical translation in the field of irisin therapy.

## 5 Experimental Section

### Preparation of MOF/MOF@irisin

The irisin solution (2 mg mL^−1^, 50 µL) was added to 2‐methylimidazole solution (3.5 mmol, 1 mL, Sanggong, China) with continuous vigorous stirring at 500 rpm for 30 min. Then, the zinc acetate aqueous solution (0.082 mmol, 0.1 mL, Aladdin, China) was slowly added into the above mixture with continuous stirring at room temperature (RT) for another 30 min. The resulting mixture was then centrifuged at 12 000 rpm for 15 minutes at a temperature of 4 °C and subsequently the precipitate was washed twice with deionized water. Finally, it was mixed with a polyvinylpyrrolidone (PVP) aqueous solution containing 3% PVP (Sigma Aldrich, USA), followed by stirring for an additional ≈30 min at RT. Pure MOF was prepared using the same method as the above procedure without the addition of irisin. The MOF or MOF@irisin NPs were stored at a temperature of 4 °C until further use.

### Preparation of MCM@MOF/MCM@MOF@irisin

The preparation of macrophage membrane‐coated nanoparticles (MCM‐NPs) was conducted following a previously reported protocol.^[^ ^23^ ^]^ Briefly, macrophage membranes (MCM) were obtained through continuous extrusion and then subsequently washed and purified with ice‐cold TM buffer containing 0.25 m sucrose. The protein content on the membrane was quantified using the bicinchoninic acid (BCA) protein assay (Beyotime Biotechnology, China). Subsequently, 1 mg of isolated cell membrane was mixed with 1 mg of MOF or MOF@irisin and extruded through 200 nm polycarbonate membranes to fabricate cell membrane‐coated MOF nanoparticles (MCM@MOF or MCM@MOF@irisin).

### Characterization of MCM@MOF/MCM@MOF@irisin

The particle sizes and zeta potential were determined using dynamic light scattering (DLS) (ZS90, Malvern, UK). To visualize the morphology of MCM@MOF, the nanoparticle sample was dropped onto a 200‐mesh carbon‐coated copper grid (Zhongjingkeyi Technology, China), stained with 1% phosphotungstic acid (Sigma Aldrich, USA), and subsequently dried for imaging on a transmission electron microscope (JEM‐2100PLUSJEOL, Japan) operating at 200 kV. The co‐localization of NBD‐labeled macrophage membrane (green) and DiD‐labeled MOF (red) was investigated by confocal laser scanning microscopy (CLSM, Olympus FluoView 1200, Japan) imaging on RAW264.7 cells incubated with MCM@MOF for 2 h.

### Drug Loading Study

The irisin encapsulation efficiency (EE) and irisin loading content (LE) of MOF were calculated using the following equations:

(1) $$EE\left(\%\right)=M_{\mathrm{irisin}}/M_{\mathrm{added}}\times 100\%$$

(2) $$LE\left(\%\right)=M_{\mathrm{irisin}}/\left(M_{\mathrm{irisin}}+M_{\mathrm{MOF}}\right)\times 100\%$$

in which *M*_irisin_ is the mass of irisin loaded in the MOF nanoparticles, *M*_added_ is the mass of added irisin and *M*_MOF_ is the mass of MOF in the MOF@irisin nanotherapeutics. The value of *M*_irisin_ was determined by the pre‐established standard curve of irisin by florescence spectra.

### In Vitro Release Profile of MCM@MOF@irisin

The release profiles of irisin‐FITC from MCM@MOF@irisin‐FITC were quantified under different concentrations of phosphate buffered saline (PBS) at pH 7.4 and 5.0. MCM@MOF@irisin (5 mg) was dispersed in PBS (5 mL) at these pH levels with continuous shaking at 37 °C, 200 rpm. At predetermined time intervals, 200 µL aliquots were collected for measurement, and an equal volume of fresh buffer was added. The cumulative release rate of irisin was determined by analyzing the fluorescence intensities in the supernatant using a FLUOstar Omega microplate reader (Germany) at excitation/emission wavelengths of 485/520 nm. Scanning electron microscopy (JSM‐7800F, JEOL, Japan) was used to observe the nanoparticle morphology at different time points.

### Proteomic Analysis

The proteomic analysis of RAW_membrane (RAW_M), MCM vesicle (MCM), and MCM@MOF was conducted at Shotgun by Zhongke New Life Biotechnology Co., Ltd (Shanghai, China). Samples were extracted with SDT lysate and denatured in boiling water bath for 10 minutes. The samples were repeatedly rinsed with the decolorizer (100 mm NH_4_HCO3/30% acetonitrile (ACN)) until the supernatant became transparent, followed by freeze‐drying. Subsequently, the samples were reduced with 10 mm dithiothreitol (DTT), alkylated with 60 mm iodoacetamide (IAA), and digested with trypsin (10 ng µL ^−1^) at 37 °C for 20 h. LCMS/MS analysis was performed on 1 µL enzymolysis products using a nanoliter flow rate HPLC liquid phase system called Easy nLC from Thermo Scientific. Briefly, the sample was loaded onto the chromatographic column Thermo scientific EASY column (2 cm×100 µm 5 µm‐C18) and separated by the analytical column Thermo scientific EASY column (75 µm×100 mm^3^ µm‐C18) at a flow rate of 300 nL min^−1^. The peptides separated by chromatography were analyzed using a Q‐Exactive mass spectrometer (Thermo Scientific, USA). Data analysis was performed using MaxQuant software version 1.6.14 and searched against the UniProtKB database.

### Western Blotting

The protein samples, RAW_M, MCM, MCM@MOF, MOF, cells, or kidney tissues were prepared to have the same overall protein concentration and denatured. Subsequently, the same amount of each sample was loaded onto a 10% sodium dodecyl sulfate polyacrylamide gel and transferred electrophoretically onto nitrocellulose filter membranes (NC). After blocking with 5% nonfat dry milk at room temperature for 1 hour, the NC membranes were incubated overnight at 4 °C with primary antibodies against CCR2 (ab273050, Abcam), CD47 (ab218810, Abcam), CD40 (28158‐1‐AP, Proteintech), CD18 (10554‐1‐AP, Proteintech), β‐actin (66009‐1‐Ig, Proteintech), SOD2 (24127‐1‐AP, Proteintech), Cytochrome C (ab133504, Abcam) and Cleaved Caspase‐3 (ab32042, Abcam). Following this step, a secondary antibody (Goat anti‐Mouse or anti‐rabbit IgG [H+L] Cross‐Adsorbed Secondary Antibody DyLight™ 800, Invitrogen) was put onto the membranes for 1 h at room temperature in the dark. Finally, the blots were imaged using a LiCor Odyssey laser scanner (LiCor Biosciences, USA).

### Cell Culture

HK‐2 cells were cultured in DMEM/F12 medium (Gibco, USA), supplemented with 10% fetal bovine serum (FBS, Biological Industries, Israel). Human umbilical vein endothelial (HUVEC) cells were cultured in RPMI‐1640 medium (Gibco, USA) containing 10% FBS. RAW264.7 cells were cultured in DMEM medium (Gibco, USA) supplemented with 10% FBS. All cell lines were incubated at 37 °C under a humidified atmosphere of 5% CO_2_.

### Cell Viability

HK‐2 cells were seeded at a density of 1×10^4^ cells per well in 96‐well plates. The cells were cultured in a humidified atmosphere containing 5% CO_2_ at 37 °C for 24 h, and then cultured under normoxic or hypoxic conditions for another 24 h. During reoxygenation, the cells were treated with PBS, irisin (100 ng mL^−1^), MCM@MOF (3.5 µg mL^−1^), and MCM@MOF@irisin with the same dose of irisin in DMEM/F12 medium containing 10% FBS. After a 6‐hour incubation, the cell viability was determined using a cell counting kit‐8 (CCK‐8, MedChemExpress, USA). The absorbance values were recorded with a FLUOstar Omega microplate reader (Germany) at 450 nm.

### Cellular Uptake

The RAW264.7 macrophage cells were seeded into 24‐well plates and incubated overnight at 37 °C. Subsequently, the media were replaced with fresh media or media supplemented with MOF@DiD or MCM@MOF@DiD, further incubated for 2 h, 8 h, and 18 h at 37 °C under a 5% CO_2_ atmosphere. Then, the cells were washed three times with PBS and fixed using a solution of 4% paraformaldehyde for 10 min at room temperature. The cells were washed three times with PBS and stained with DAPI for 10 min. Cellular observations were conducted using CLSM.

### In Vitro Binding of MCM@MOF

HUVECs were seeded onto coverslips in 24‐well plates and incubated for 24 h. The cell culture medium was replaced, and the pro‐inflammatory cytokine TNF‐α (50 ng mL^−1^, Sigma Aldrich, USA) was added to the culture. After a 6‐hour stimulation period, the cells were treated with MCM@MOF@DiD at 4 °C for 2 h, followed by three washes with PBS. Subsequently, the cells were fixed with 4% paraformaldehyde for 10 minutes at room temperature and blocked using quick blocking solution (Beyotime Biotechnology, China). The primary antibody against rabbit anti‐ICAM‐1 (60299‐1‐Ig, Proteintech, China) was then incubated with the cells overnight at 4 °C. Following incubation, the cells were washed three times with PBS before staining with DAPI and imaged using CLSM.

### Animal Model

Eight‐week‐old C57BL/6J mice were provided by the Laboratory Animal Center of the Daping Hospital (Chongqing, China). Following a week of acclimation to the environment with normal feed, they were randomly assigned to different experimental groups. The mouse model of renal ischemia reperfusion injury was established as previously described.^[^ ^31^ ^]^ Briefly, the mice were anesthetized by the intraperitoneal injection of 1% pentobarbital. A mid‐abdominal incision was made to expose the kidney while ensuring that warm and moistened gauze covered the intestines to prevent desiccation. Subsequently, a right nephrectomy was performed followed by clamping of the left renal pedicle for 45 min using a non‐traumatic microvascular clip. After releasing the clamp, 1 mL sterile saline was subcutaneously injected for rehydration. The incision was sutured after observing a color change in the kidney from purple‐black to red. Mice undergoing similar surgical procedures without renal pedicle clamping served as sham controls. Throughout surgery, the mice were maintained on a heating pad at 37 °C to maintain constant body temperature.

### Interaction of Macrophage Membrane Proteins With the Receptors in the Damaged Kidney

Gene‐level mRNA expression data after renal ischemia‐reperfusion injury were available from Nephroseq (www.nephroseq.org). To analyze whether macrophage membrane proteins can target the damaged kidney by binding to their receptors, protein–protein interactions were sourced from BioGrid (http://thebiogrid.org/).

### In Vivo Biodistribution of MCM@MOF@irisin

The unilateral renal ischemia‐reperfusion injury mouse model was established as previously described.^[^ ^31^ ^]^ 0.2 mL of irisin‐Cy5.5 and MCM@MOF@irisin‐Cy5.5 were administered via intravenous injection (i.v.) at a constant dosage of irisin (10 µg mL^−1^), respectively. Saline was injected as controls using the same dose. At different time points, the mice were anesthetized and imaged using an in vivo imaging system (IVIS, PerkinElmer, USA). Subsequently, the mice were sacrificed and vital organs including the heart, lung, liver, spleen, brain, and kidney were collected for tissue distribution analysis at 2 h and 24 h post‐administration. Fluorescence images were obtained using the IVIS spectrum imaging system with an excitation wavelength of 670 nm and an emission wavelength of 690 nm. Fluorescence intensity was calculated after subtracting the saline background signal. Moreover, frozen kidney sections were observed by CLSM.

### Pharmacokinetics Studies

C57BL/6J mice aged 8 weeks were intravenously injected with 200 µL of saline, or MOF (20 mg kg^−1^), or MCM@MOF (20 mg kg^−1^). At different time points post‐injection, 50 µL of blood was collected from the tail vein. Nitric acid and H_2_O_2_ (30%) were subsequently added to the collected blood, followed by heating at 150 °C until the solution became clear and transparent. The quantification of Zn content was performed using inductively coupled plasma atomic emission spectrometry (ICP‐AES, Agilent 7700x, USA).

### Mitochondria Targeting Ability

HK‐2 cells were seeded on 24‐well plates with glass coverslips and incubated at 37 °C, 5% CO_2_ overnight. For the hypoxia/reoxygenation group, the cells were exposed to hypoxic conditions (1% O_2_, 94% N_2_, and 5% CO_2_) for 24 h in serum‐free DMEM/F12 medium, followed by reoxygenation under normoxic conditions (5% CO_2_ and 95% air) for 6 h in normal DMEM/F12 medium or media supplemented with irisin‐Cy5.5 (100 ng mL^−1^), or MCM@MOF@irisin‐Cy5.5 with the same dose of irisin. After washing, the mitochondria were stained with MitoTracker Red CMXRos (Beyotime Biotechnology, China) at a concentration of 200 nm for 30 min at 37 °C. The nuclei were stained with DAPI for 10 min. The fluorescence images of the cells were conducted by CLSM.

### Mitochondrial Membrane Potential

The HK‐2 cells were cultured on coverslips in 24‐well plates for 24 h, followed by incubation under normoxic or hypoxic conditions for an additional 24 hours. During reoxygenation, the cells were treated with irisin (100 ng mL^−1^), MCM@MOF (3.5 µg mL^−1^), and MCM@MOF@irisin with the same dose of irisin, for 6 h in DMEM/F12 medium supplemented with 10% FBS. Subsequently, 0.5 mL JC‐1 dyeing working solution (Beyotime Biotechnology, China) was added to the cell culture medium and incubated at 37 °C for 20 min to measure the relative mitochondrial membrane potential. Live cells were visualized using a fluorescence microscope (DFC7000T, Leica, Germany). JC‐1 aggregates (red fluorescence) were detected at an excitation/emission wavelength of 525/590 nm, while JC‐1 monomers (green fluorescence) were detected at an excitation/emission wavelength of 490/530 nm. The relative mitochondrial membrane potential was quantified by calculating the ratio of red/green fluorescence intensity (590/530 nm).

### Measurement of Intracellular ROS Levels

HK‐2 cells were seeded in 24‐well plates and stimulated with hypoxia for 24 h. Subsequently, the cells were incubated with PBS, irisin, MCM@MOF, and MCM@MOF@irisin in DMEM/F12 medium containing 10% FBS for 6 hours. Following treatment, the culture media was replaced with DMEM/F12 medium containing 10 µM DCFH‐DA fluorescent probe (Beyotime, China) and incubated at 37 °C for 20 min. The cells were then washed three times with DMEM/F12 medium and observed using a fluorescence microscope (DFC7000T, Leica, Germany).

### Oxidative Stress

The HK‐2 cells were subjected to H/R stimulation for 24 h and then treated with irisin, MCM@MOF, or MCM@MOF@irisin for 6 h. Kidney samples for determination of MDA, CAT activity, and total SOD activity were obtained 24 h after renal I/R injury. The cells and kidneys were lysed at 4 °C and centrifuged at 12 000 rpm for 10 min, followed by collection of the supernatants. Total SOD activity was measured using Total SOD Assay Kit (Beyotime Biotechnology, China). CAT activity was measured by Catalase Assay Kit (Beyotime Biotechnology, China), while MDA levels were measured using a thiobarbituric acid reactive substances assay kit (Beyotime Biotechnology, China).

### In Vivo Treatment Efficacy Studies

The mouse renal I/R injury model was established as previously described.^[^ ^31^ ^]^ The mice were randomly divided into five groups: sham, I/R, and three treatment groups (I/R with irisin, I/R with MCM@MOF, I/R with MCM@MOF@irisin). Mice in the treatment group received intravenous 200 µL of irisin or MCM@MOF@irisin at a dose of 5 µg mL^−1^, MCM@MOF at a dose of 175 µg mL^−1^ immediately after I/R injury. The sham group underwent the same surgical procedure without ischemia. Both the sham group and model group received an equivalent volume of saline as controls. After 24 h of treatment, the mice were euthanized, and blood and kidneys were collected for subsequent analysis. For analysis of the effects of irisin and MCM@MOF@irisin on the progression from AKI to CKD, mice subjected to unilateral I/R injury received 200 µL of irisin or MCM@MOF@irisin (1 µg mL^−1^) via the tail vein once every two days for one week and euthanized at 4 weeks after reperfusion. The kidneys were collected for further analysis.

### Renal Function and Histology

The levels of serum creatinine and blood urea nitrogen were measured using a commercially available kit (Nanjing Jiancheng Biotech, China). The renal tissues were perfused with PBS, fixed in 4% paraformaldehyde, embedded in paraffin, sectioned to 4‐µm thickness, and stained with periodic acid–Schiff (Solarbio, China). Ten random tissue sections per mouse were evaluated for tubular injury score based on semiquantitative assessment of tubular rupture, dilation, rupture, and cast formation. The scoring ranged from 0 to 5 as follows: 0, no lesion; 1, <10%; 2, 10 to 25%; 3, 26 to 50%; 4, 51 to 75%; 5, >75%. Renal fibrosis was detected by Sirius red staining (Sigma Aldrich, USA) and the area of fibrosis was measured from ten randomly selected sections per mouse.

### TUNEL Assay

The kidneys were harvested post‐I/R and treatment, fixed overnight at 4 °C in 4% paraformaldehyde, dehydrated, made transparent, wax‐embedded, and sectioned. Subsequently, the sections were subjected to cell apoptosis staining using the terminal deoxynucleotidyl transferase (TdT)‐mediated dUTP nick‐end labeling (TUNEL) Kit (Beyotime Biotechnology, China), following the manufacturer's instructions. The nuclei were further stained with DAPI for 10 min. Fluorescence staining was viewed under a fluorescence microscope (Olympus, Japan).

### Immunofluorescence Staining

The kidneys were harvested from euthanized mice after reperfusion. The kidneys were fixed in 4% paraformaldehyde, dehydrated, embedded in paraffin, and sectioned into 4‐µm slices. Following deparaffinization and rehydration, the sections underwent a 20‐minute antigen retrieval process in citrate acid buffer (pH 6.0) at 95 °C. Subsequently, the tissues were washed three times for 5 min with PBS and then blocked with immunostaining blocking buffer (Beyotime Biotechnology, China) for 30 min at room temperature. The sections were incubated overnight at 4 °C with mouse TIM‐1/KIM‐1/HAVCR antibody (1:200, R&D Systems, USA), α‐SMA (1:200, Sigma Aldrich, USA) and COL‐1 (1:400, Proteintech, China). Alexa Fluor 488‐conjugated anti‐mouse secondary antibodies (1:200, Invitrogen, USA) were used as the secondary antibody. After the washing steps were completed, the nuclei were stained with DAPI for 10 minutes followed by imaging under a fluorescence microscope. Fluorescence intensity of KIM‐1, α‐SMA, and COL‐1 were quantified as the mean intensity per pixel area.

### Quantitative Real‐Time PCR Assay

Total RNA was isolated from frozen kidneys using TRIzol reagent (TaKaRa, Japan). Briefly, the kidneys were homogenized in TRIzol, followed by the addition of chloroform. The upper aqueous phase containing RNA was collected and mixed with an equal volume of isopropanol to precipitate the RNA. Subsequently, the RNA pellet was washed twice with 75% ethanol and dissolved in RNase‐free water. Reverse transcription of RNA into complementary DNA (cDNA) was performed using a Reverse Transcription Kit (TaKaRa, Japan). Quantitative real‐time PCR (qPCR) was then conducted using SYBR Green Master MIX (Invitrogen, USA) and gene‐specific primers. Relative mRNA levels were determined using the comparative threshold (CT) cycle method and normalized to GADPH expression. The primer sequences are provided in Table S1 (Supporting Information).

### Enzyme‐Linked Immunosorbent Assay (ELISA)

Kidneys were collected and lysated for measuring TNF‐α, IL‐1β, CCL‐2, and IL‐6 levels according to per manufacturer instructions (Mouse TNF‐alpha ELISA Kit, KE10002, Proteintech; Mouse IL‐1 beta ELISA Kit, KE10003, Proteintech; Mouse MCP‐1 ELISA Kit, KE10006, Proteintech; Mouse IL‐6 ELISA Kit, KE10007, Proteintech).

### Bio‐Transmission Electron Microscopy (Bio‐TEM)

The renal tissues were quickly cut into 1 mm^3^ pieces and placed in an electron microscope fixative (Servicebio, China) for 2 h at room temperature in the dark. Then, they were fixed at 4 °C for preservation and transportation. The tissues were then post‐fixed in a solution of 1% osmium tetroxide, dehydrated using graded ethanol series, and embedded in resin. Ultra‐thin sections (60‐80 nm) were cut from the resin blocks and collected onto the 150 meshes cuprum grids. Uranyl acetate and lead citrate were used to stain the sections. Finally, the cuprum grids were observed under TEM (HT7700, Hitachi, Japan) to capture images.

### DHE Staining

The fresh kidney samples were embedded in Tissue‐Tek O.C.T compound (Sakura, USA) and snap‐frozen. Subsequently, 8‐µm sections were cut, air‐dried, and rinsed with PBS buffer. Then, the sections were incubated with dihydroethidium (DHE, Beyotime Biotechnology, China) for 30 min at 37 °C in the dark. After three washes with PBS, the samples were fixed using 4% paraformaldehyde and stained with DAPI before being imaged under a fluorescence microscope. All sections underwent simultaneous processing under identical conditions.

### Mitochondrial Respiratory Chain Complex Enzyme Activity Assay

Complex I and II enzymatic activities were assessed using the Complex Enzyme Activity Assay Kit (ab109721, ab109908, Abcam) following the manufacturer's protocol. Briefly, samples at a final concentration of 0.2 mg mL^−1^ proteins were loaded onto plates for the detection of Complex enzymatic activities. The measurements were performed using a FLUOstar Omega microplate reader (Germany) at 450 nm for complex I and 600 nm for complex II, respectively.

### Safety Evaluation

The long‐term and acute toxicities of the mice treated with MOF or MCM@MOF were assessed. In the case of acute toxicity assessment, the mice received a high dose of 10 mg kg^−1^ and then sacrificed after 24 h to evaluate the acute effects. For long‐term toxicity evaluation, the mice were intravenously injected with MOF or MCM@MOF at a dose of 1 mg kg^−1^ every 2 days for a duration of 21 days. After a treatment period of three weeks, all mice were euthanized, and blood samples along with major organs (heart, liver, spleen, lungs, and kidneys) were collected for histological analysis. Furthermore, the safety profile of both MOF and MCM@MOF was evaluated by analyzing blood serum using blood chemistry analysis techniques. Mice receiving an equivalent volume of saline served as controls.

### Statistical Analysis

All experiments were performed at least three times. One‐way analysis of variance (ANOVA) with Tukey's post hoc test was used for statistical analysis. All data were presented as the mean ± standard deviations (SD). P values less than 0.05 were considered statistically significant.

### Ethics Statement

All animal experiments conducted in this study received approval from the Laboratory Animal Welfare and Ethics Committee of the Third Military Medical University (AMUWEC20219012).

## Conflict of Interest

The authors declare no conflict of interest.

## Supporting information

Supporting Information

## Data Availability

The data that support the findings of this study are available from the corresponding author upon reasonable request.
