# Supplementary material for: Irisin‐Encapsulated Mitochondria‐Targeted Biomimetic Nanotherapeutics for Alleviating Acute Kidney Injury
Source: Adv Sci (Weinh). 2024 Aug 9;11(38):2402805. doi: 10.1002/advs.202402805 (PMC11481180; doi:10.1002/advs.202402805)
Supplement: Supplementary file 1 — Supporting Information [file ADVS-11-2402805-s001.docx]

Supporting Information

Irisin-encapsulated Mitochondria-Targeted Biomimetic Nanotherapeutics for Alleviating Acute Kidney Injury

*Xia Zhang^1,3,4†^, Lijia Liang^2,3,5†*^, Fengxian Wang^1,3,4^, Pedro A. Jose^6^, Ken Chen^1,3,4*^, Chunyu Zeng^1,2,3,4*^*

^1^Department of Cardiology, Daping Hospital, Third Military Medical University (Army Medical University), Chongqing, 400042, P.R. China;

^2^Chongqing Institute of Green and Intelligent Technology, Chinese Academy of Sciences, Chongqing, 400714, P.R. China;

^3^Key Laboratory of Geriatric Cardiovascular and Cerebrovascular Disease Research, Ministry of Education of China, Chongqing, 400042, P.R. China;

^4^Chongqing Key Laboratory for Hypertension Research, Cardiovascular Clinical Research Center, Chongqing Institute of Cardiology, Chongqing, 400042, P.R. China;

^5^Chongqing General Hospital, Chongqing, 401147, P.R. China;

^6^Division of Renal Diseases & Hypertension, Department of Medicine and Pharmacology-Physiology, The George Washington University School of Medicine & Health Sciences, Washington D.C., 20037, USA.

*Corresponding to: Lijia Liang, E-mail address: [lianglj@cigit.ac.cn;](mailto:lianglj@cigit.ac.cn;)

Ken Chen, E-mail address: chenken@tmmu.edu.cn;

Chunyu Zeng, MD, PhD, FACC, E-mail address: zengchunyu@tmmu.edu.cn

†These authors contributed equally to this work.


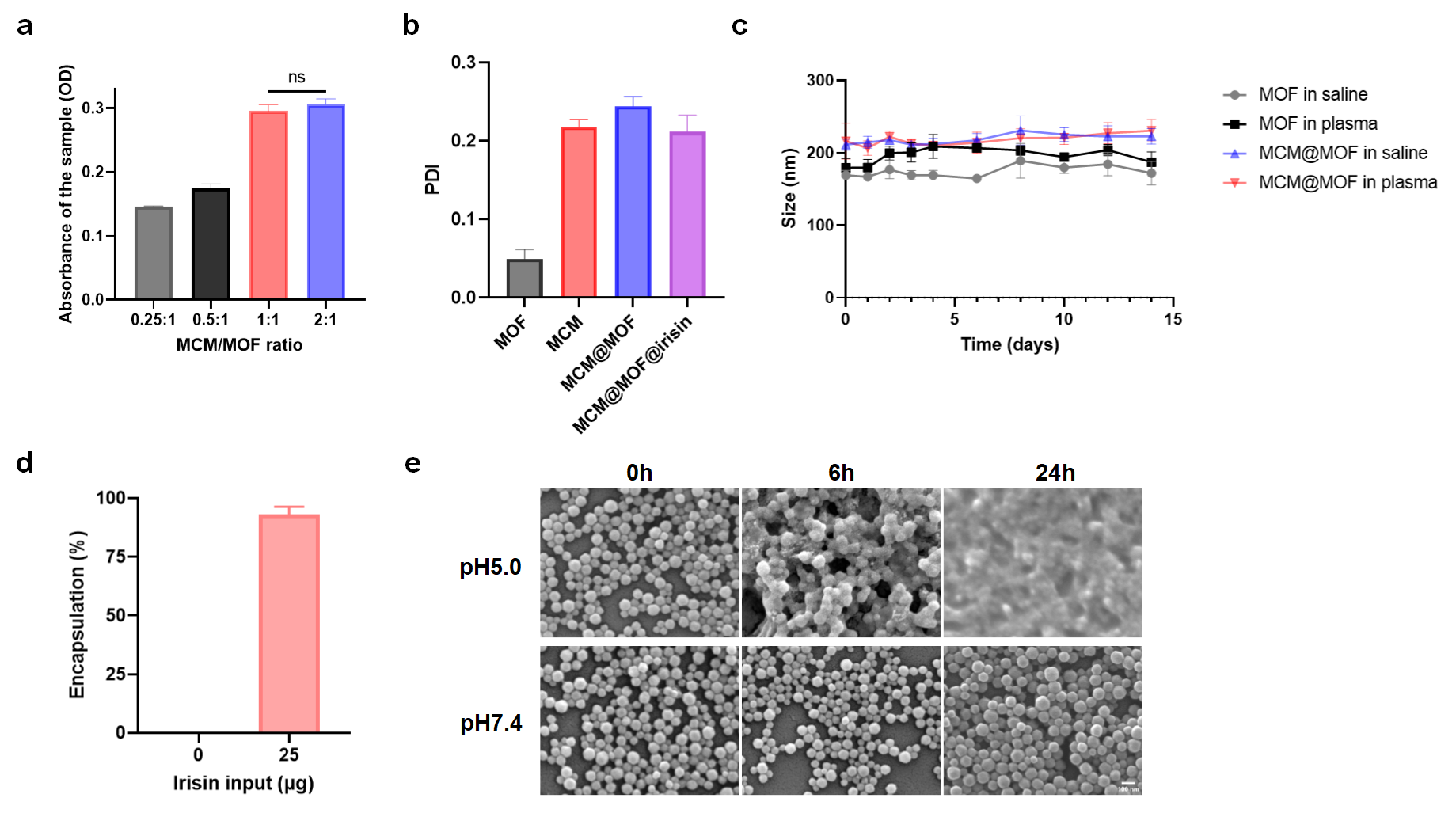


Figure S1. (a) The mass ratio of absorbance of macrophage membrane to MOF (n = 3 per group). (b) The PDI values of MOF, MCM, MCM@MOF and MCM@MOF@irisin.

(c) Stability of MOF and MCM@MOF over the course of 14 days in plasma and saline (n = 3 per group). (d) Encapsulation efficiency of irisin in MCM@MOF@irisin (n = 3 per group). (e) Representative SEM images of MOF immersed in two different pH buffers at different time points. Scale bar = 100 nm. The data are presented as mean ± SD. ns, not significant.


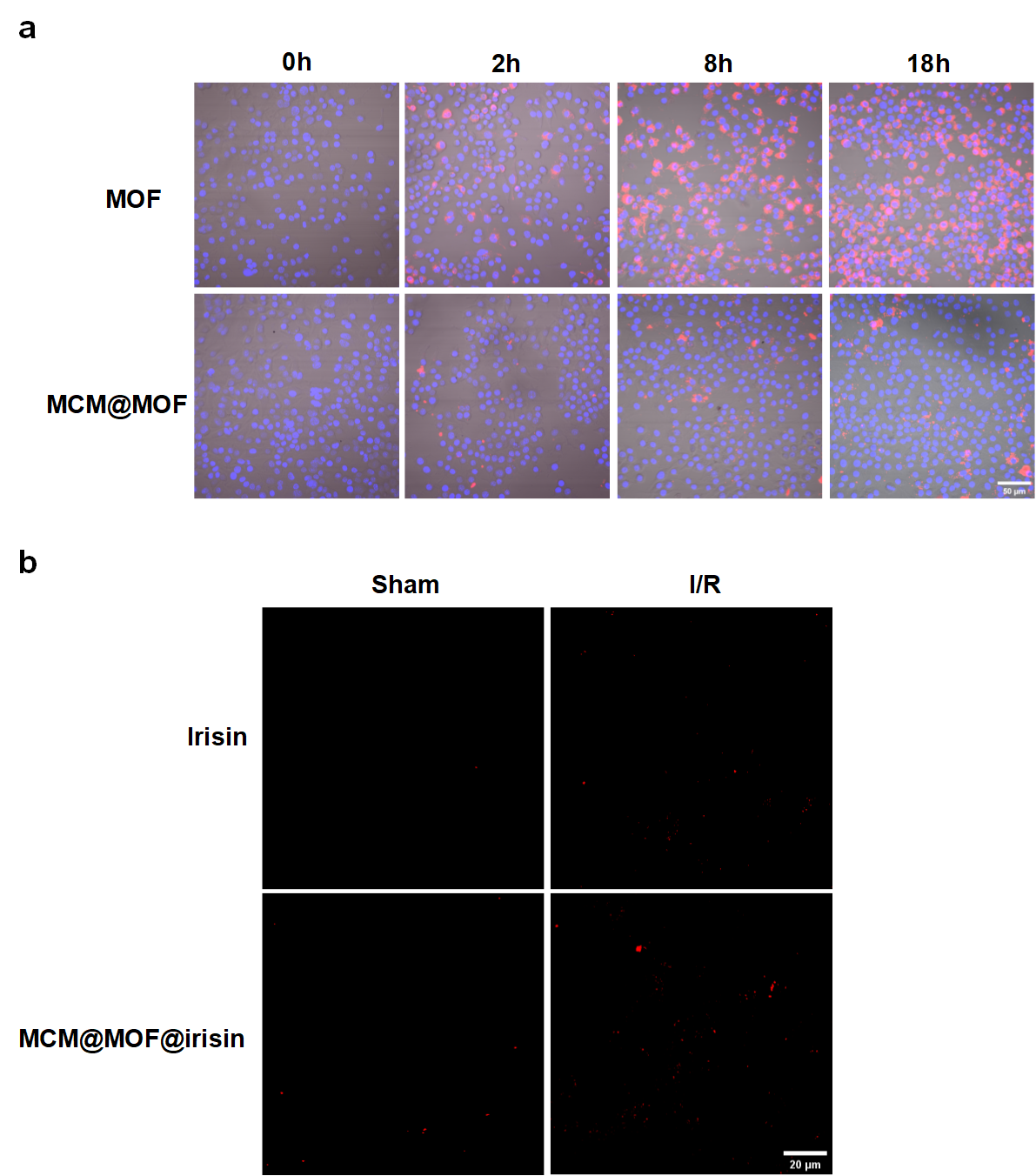


Figure S2. (a) Intracellular uptake of MOF and MCM@MOF in RAW264.7 cells after 2 h, 8 h, and 18 h incubation (n = 6 per group). Red represents nanoparticles and blue represents nuclei. Scale bar = 50 μm. (b) Representative CLSM images of accumulated irisin in kidney sections at 24 h after administration of irisin-Cy5.5 and [MCM@MOF@irisin-Cy5.5](mailto:MM@MOF@irisin-Cy5.5) (n = 3 per group). Red represents irisin. Scale bar = 20 μm.


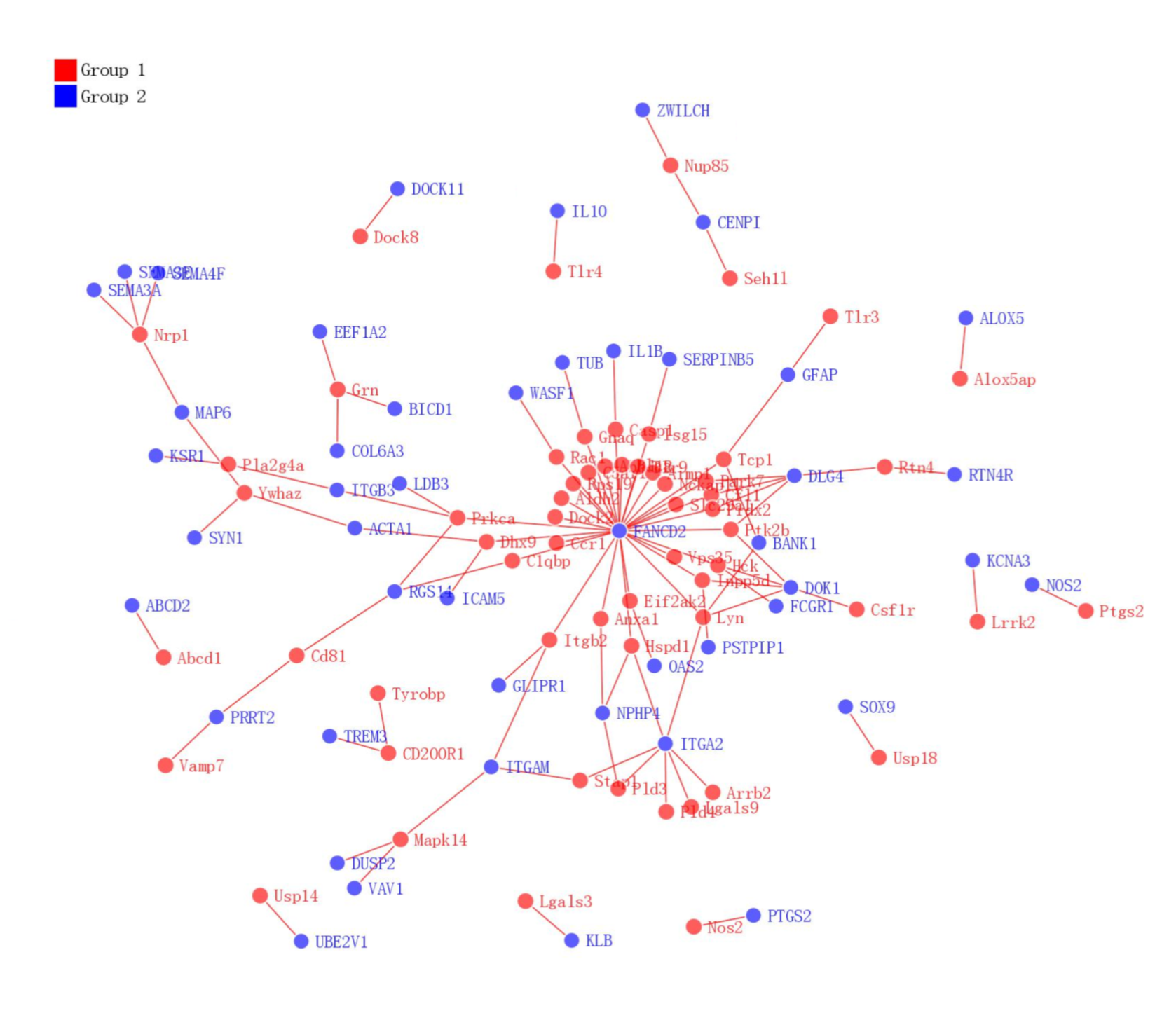


Figure S3. The interactions between the proteins related to inflammation and chemotaxis on macrophage membrane and the receptors in the damaged kidney. Group1: the proteins related to inflammation and chemotaxis on the macrophage membrane; Group 2: the receptors whose levels increased after kidney injury.


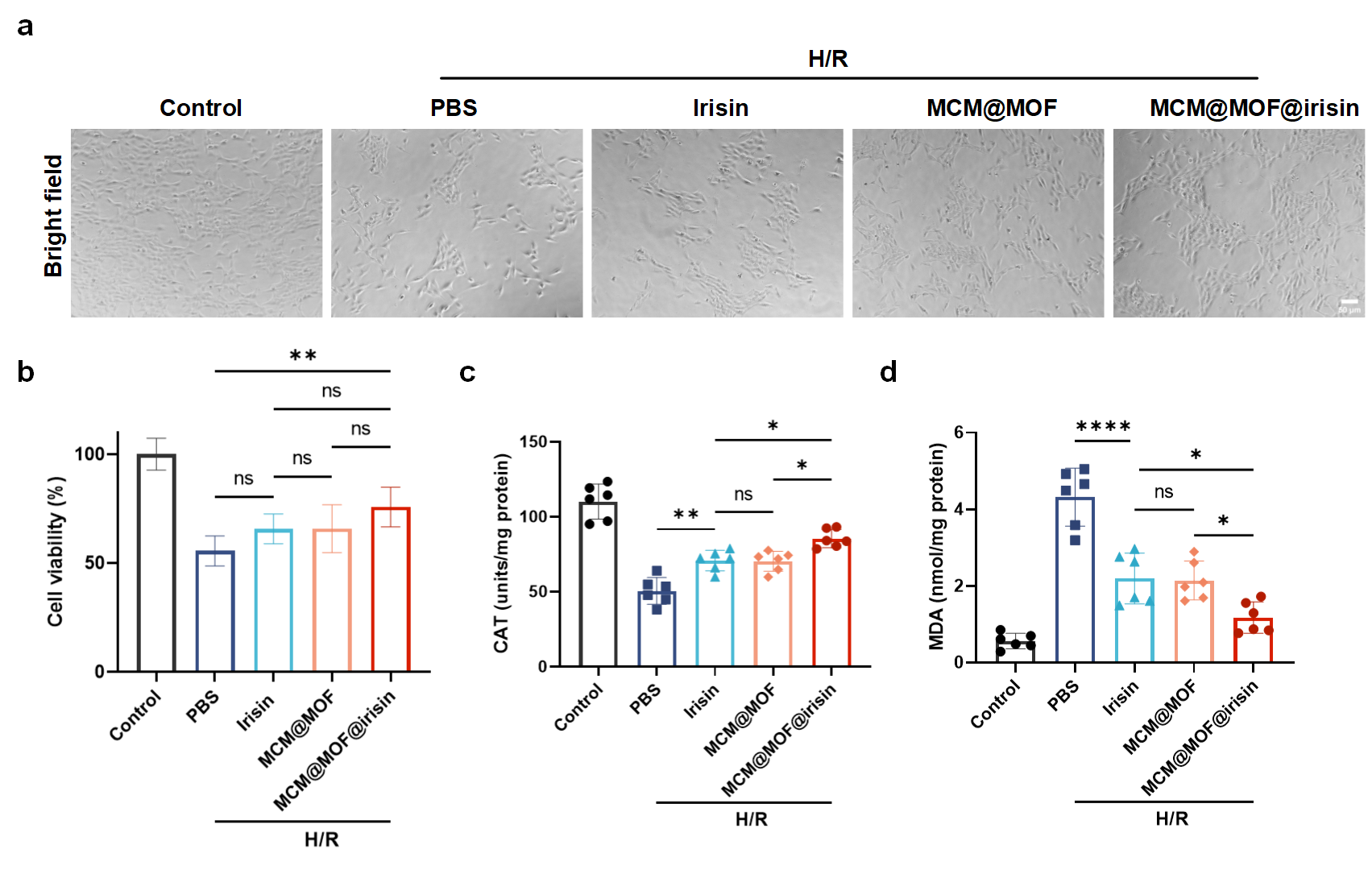


Figure S4. (a) Representative optical micrographs of HK-2 cells with different treatments (n = 6 per group). Scale bar = 50 μm. (b) Cell viability of HK-2 cells incubation with PBS, irisin, MCM@MOF or MCM@MOF@irisin after hypoxia (n = 6 per group). (c) CAT activities of H/R injured HK-2 cells after incubation in PBS, irisin, MCM@MOF or MCM@MOF@irisin; HK-2 cells incubated with PBS under normoxia served as control (n = 6 per group). (d) Intracellular MDA levels in the HK-2 cells after the different treatments (n = 6 per group). The data presented as mean ± SD. * P ＜ 0.05, ** P ＜ 0.01, **** P ＜ 0.0001, ns, not significant.


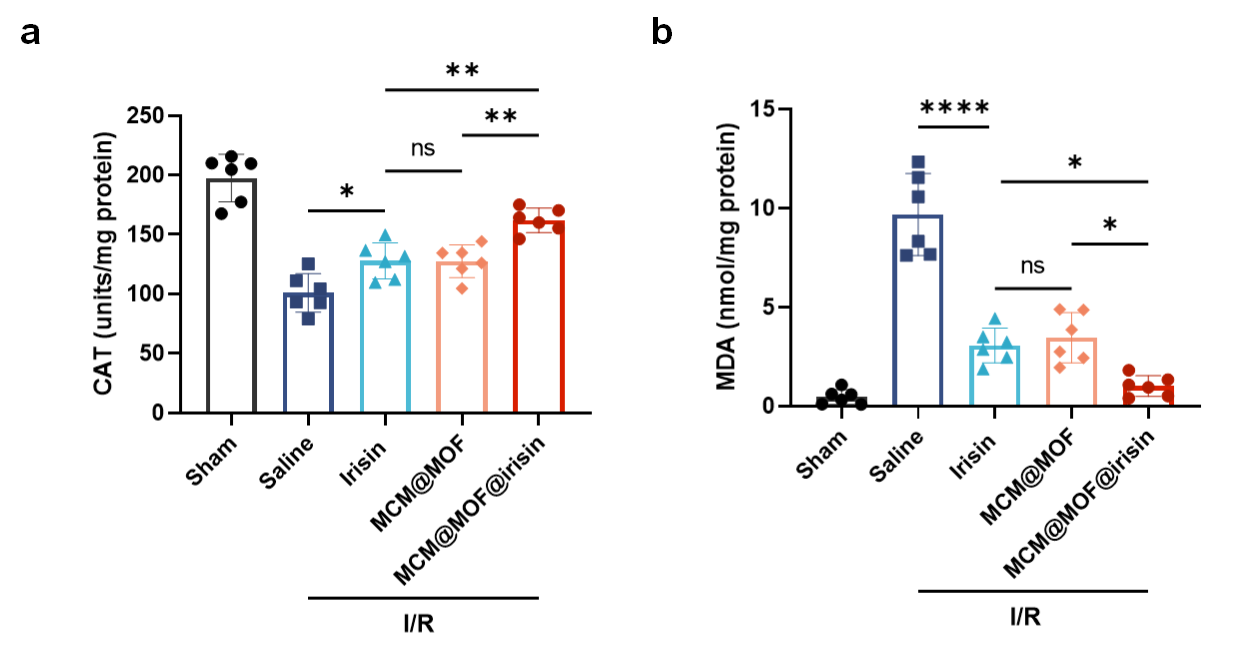


Figure S5. (a) CAT activities of kidneys after treatment with saline, irisin, MCM@MOF or MCM@MOF@irisin (n = 6 per group). (b) MDA levels in kidneys from saline-, irisin-, MCM@MOF- and MCM@MOF@irisin-treated mice (n = 6 per group). The data presented as mean ± SD. * P ＜ 0.05, ** P ＜ 0.01, **** P ＜ 0.0001, ns, not significant.


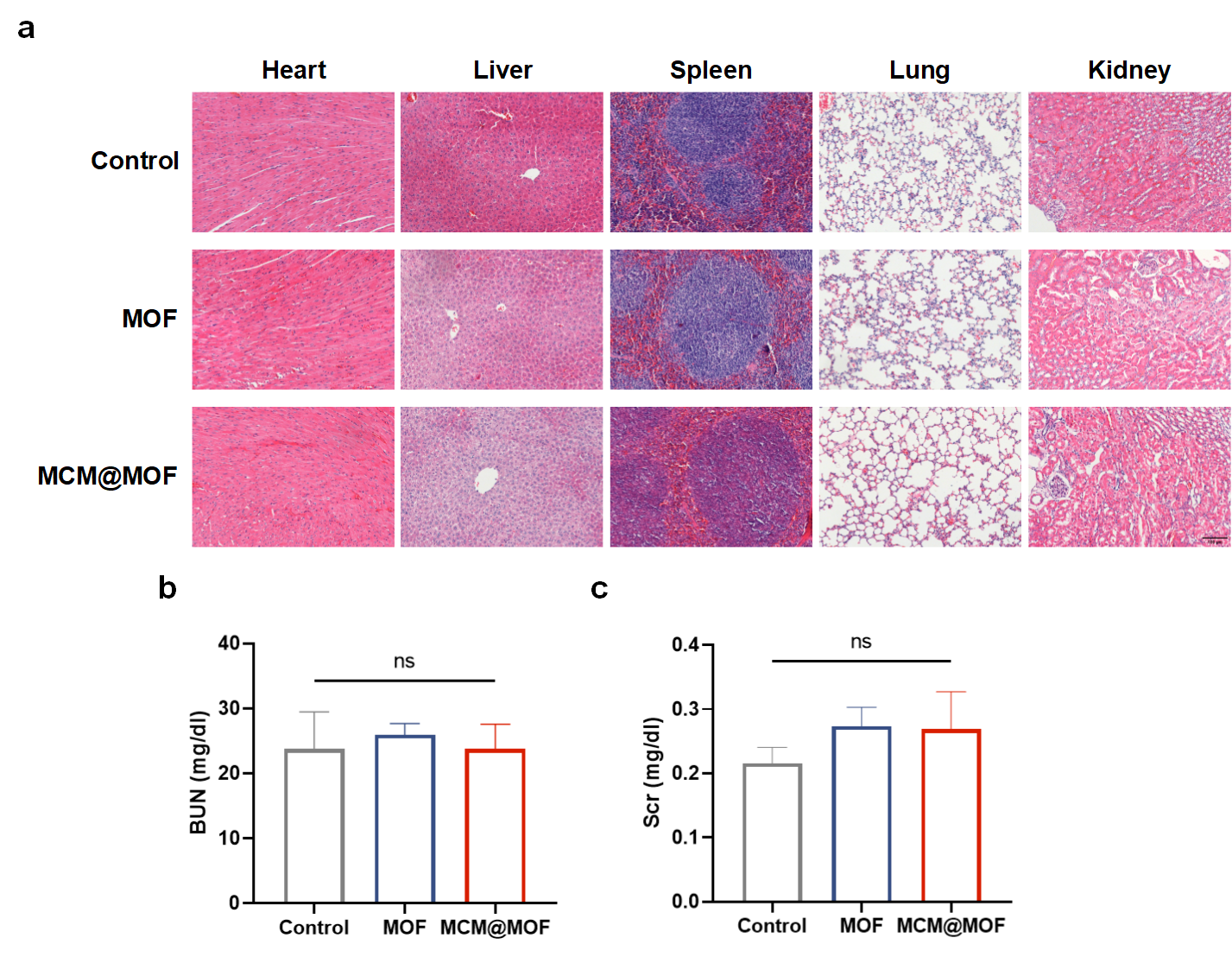


Figure S6. (a) H&E-stained sections of major organs 24 h after the intravenous administration of saline, MOF, or MCM@MOF into healthy mice (n = 3 per group). Scale bar = 100 μm. (b) BUN and (c) serum creatinine levels in mice injected with saline, MOF, or MCM@MOF (n = 3 per group). The data are presented as mean ± SD. ns, not significant.

Figure S7. mRNA expression of SOD2 in I/R kidney lysates from each group (n = 6 per group). GADPH served as a loading control. The data presented as mean ± SD. * P ＜ 0.05, ** P ＜ 0.01, ns, not significant.

Supplementary Table S1

| Gene | Forward primer (5′ to 3′) | Reverse primer (5′ to 3′) |
| --- | --- | --- |
| IL-6 | ACAGAAGGAGTGGCTAAGGA | AGGCATAACGCACTAGGTTT |
| IL-1β | GGTAAGTGGTTGCCCATCAGA | GTCGCTCAGGGTCACAAGAAA |
| CCL-2 | ACCTTTTCCACAACCACCT | GCATCACAGTCCGAGTCA |
| GADPH | TGGCTACAGCAACAGGGT | TTATGGGGTCTGGGATGG |
| TNF-α | CGCTGAGGTCAATCTGC | GGCTGGGTAGAGAATGGA |
| SOD2 | GCCTGCTCTAATCAGGACCC | TAGTAAGCGTGCTCCCACAC |

Table S1: Primer sequences for quantitative real-time PCR.
